# Assessment of hydrogen transport aircraft: Effects of fuel tank integration

**DOI:** 10.1007/s13272-022-00601-6

**Published:** 2022-09-17

**Authors:** G. Onorato, P. Proesmans, M. F. M. Hoogreef

**Affiliations:** Faculty of Aerospace Engineering, Delft University of Technology, P.O. Box 5058, 2600 GB Delft, The Netherlands

**Keywords:** Aircraft Design, Hydrogen Aircraft, Fuel tank integration

## Abstract

Zero-carbon-dioxide-emitting hydrogen-powered aircraft have, in recent decades, come back on the stage as promising protagonists in the fight against global warming. The main cause for the reduced performance of liquid hydrogen aircraft lays in the fuel storage, which demands the use of voluminous and heavy tanks. Literature on the topic shows that the optimal fuel storage solution depends on the aircraft range category, but most studies disagree on which solution is optimal for each category. The objective of this research was to identify and compare possible solutions to the integration of the hydrogen fuel containment system on regional, short/medium- and large passenger aircraft, and to understand why and how the optimal tank integration strategy depends on the aircraft category. This objective was pursued by creating a design and analysis framework for CS-25 aircraft capable of appreciating the effects that different combinations of tank structure, fuselage diameter, tank layout, shape, venting pressure and pressure control generate at aircraft level. Despite that no large differences among categories were found, the following main observations were made: (1) using an integral tank structure was found to be increasingly more beneficial with increasing aircraft range/size. (2) The use of a forward tank in combination with the aft one appeared to be always beneficial in terms of energy consumption. (3) The increase in fuselage diameter is detrimental, especially when an extra aisle is not required and a double-deck cabin is not feasible. (4) Direct venting has, when done efficiently, a small positive effect. (5) The optimal venting pressure varies with the aircraft configuration, performance, and mission. The impact on performance from sizing the tank for missions longer than the harmonic one was also quantified.

## Introduction

In line with the 2015 Paris Agreement, the Air Transport Action Group (ATAG) has set the goal of reducing the aviation’s net $$\hbox {CO}_2$$ emissions to 50% of their 2005 levels by 2050.^1^$$^{,}$$^2^ In the last 30 years, a combination of technology developments in aircraft engines and airframes, in the way these aircraft are operated, in the infrastructure environment and the utilisation of assets has brought a 54% decrease in $$\hbox {CO}_2$$ emissions per passenger per km $$^{1}$$. Nevertheless, in the same period, demand for air travel has more than tripled^3^ and is forecast to increase by 3–4% per year until 2050. $$^{1,2,}$$^4^ Even if efficiency improvements would accelerate from 1.5 to 2% per year, as targeted by ICAO (International Civil Aviation Organization), this growth would cause $${\hbox {CO}_2}$$ emissions from aviation to double instead of halve by 2050. Therefore, despite the stagnant growth due to the COVID-19 crisis$$^{1}$$, more decisive decarbonisation measures will be required to respect the Paris Agreement and to meet the ATAG target.

Considering these stringent targets, hydrogen is recognised to have a fundamental advantage over kerosene in terms of climate impact. Indeed, either through direct combustion in gas turbines or via conversion in fuel cells, hydrogen has zero direct ${\hbox {CO}_2}$ emissions and low (or zero, in case of fuel cells) $\hbox {NO}_x$ emissions [1]. Moreover, despite ${\hbox {H}_2}$ turbines (and fuel cells) emitting 2.55 times the water vapour of kerosene ones, the increase in the annually averaged global-mean radiative forcing of ${\hbox{H}}_{2}{\hbox{O}}$ from 0.9 to 2.6 mW/m^2^ (in 2015) [2, 3] would be insignificant when compared to the 38 mW/m^2^ radiative forcing of ${\hbox{CO}}_2$ which would be eliminated [3]. Currently, it is estimated that the largest contribution to the aviation-induced radiative forcing (55% [4, 5]) comes from the aircraft-induced cloudiness (AIC). Despite ${\hbox {H}_2}$ turbines producing more water vapour and causing the contrails to form more frequently, they emit fewer aerosol particles compared to kerosene ones. Because of this, ${\hbox {H}_2}$ aircraft contrails are characterised by fewer and larger ice crystals, which lower the optical depth of contrails and reduce the induced radiative forcing [6, 7]. Overall, with ${\hbox {H}_2}$ aircraft, a contrail-related climate impact reduction between 16% and 29% in terms of radiative forcing could be achieved, according to the 2015–2050 transition scenarios [8]. More recently, Burkhardt et al. further studied the potential reduction in radiative forcing as a function of the initial ice crystal number.

Research on the application of hydrogen as an alternative aviation fuel dates back several decades. Hans von Ohain (hydrogen-powered turbojet engine HeS 1, in 1937^5^), NACA (in 1955 [9, 10]), the US Air Force (B-57 on hydrogen fuel, in 1957^6^), Lockheed (Lockheed CL-400 Suntan, in the 1950s^7^), the Soviet Union (Tupolev Tu-155 Laboratory aircraft, in 1988 [11]), and projects in Europe (in the 1990s,^8^) recognised the attractive combustion characteristics and the high specific energy of hydrogen. Yet, these two hydrogen qualities alone had, evidently, not been enough to outweigh the disadvantages, such as its low energy density and its difficult handling and storage, thus hydrogen planes never went past the experimental phase. In the last decades, however, investigations into the safety of handling and air transport of $\text {L}\text {H}_\text {2}$ found that the hydrogen aircraft are not more dangerous than kerosene ones [12, 13] and this, coupled with the need for reduced aviation emissions, prompted the funding of several government-backed studies, in the field of both hydrogen-combustion-powered^9^ and fuel-cell-powered [14] aircraft.

Most recently, in September 2020, Airbus revealed its plan to develop three new aircraft concepts integrating direct combustion of hydrogen through a modified gas turbine featuring an embedded electric motor powered by fuel cells. These concept aircraft are expected to shape the development of Airbus’ future zero-emission aircraft.^10^ Airbus’ solution to the tank integration problem for these concepts consists of placing the tank in the aft fuselage section by increasing the length and diameter of the latter.^11^ Despite that the tank integration solution envisioned by Airbus appears to be straightforward, the introduction of a large, in-flight centre-of-gravity (c.g.) variation causes most studies to disagree on the optimality or even feasibility of this solution for other than short-range airliners. The Cryoplane Project$^{9}$ relegated the adoption of a single, aft tank to small regional (REG) aircraft, and only in combination with artificial stability systems. The McKinsey & Company study$^{2}$ set the size limit for the aft tank layout to the short-range aircraft category (165 passengers, 2000 km range). Verstraete et al. [15] investigated this tank option for a REG airliner and excluded it from the design of large passenger aircraft (LPA). Troeltsch found that the combination of forward and aft tanks was the most efficient for its LPA [16]. Brewer, who in his book collected the accumulated knowledge of hydrogen aircraft technology up to his time, did not investigate the single aft tank option [17]. Maniaci, who conceptualised an $\text {L}\text {H}_\text {2}$ transport aircraft starting from a Boeing 747-400, selected the non-c.g.-critical overhead tank integration solution [18]. The Tupolev Tu-155 was designed to replace the medium-range Tu-154 but could only carry enough hydrogen in its single aft tank to fly a short-range mission (1700 km with 90 passengers).^12^ The single study in which the aft tank configuration is used on a short/medium-range (SMR) aircraft appears to be the recent study conducted by Silberhorn et al. [19], which concluded that for a 5741 km mission with 165 passengers, a hydrogen version of the aircraft would have a 3.5% lower maximum take-off mass, 11% higher operational empty mass and a 7% higher specific energy consumption than its kerosene counterpart.^13^ Nevertheless, the seemingly unvaried horizontal tail size of the $\text {L}\text {H}_\text {2}$ aircraft with respect to the kerosene baseline highlights the possibility that the inevitable increase in the centre-of-gravity range had not been properly taken into account.

The foregoing discussion reveals a lack of consistency among the conclusions of these studies. Multiple reasons for this issue are identified: (1) the technology levels considered belong to different years, (2) the design assumptions differ between the studies, leading to different sizing processes and (3) the design and analysis frameworks used by the various authors are of different levels of fidelity.

To overcome these hurdles, the objective of this research was to identify and compare possible solutions for the integration of the liquid hydrogen fuel system on REG, SMR and LPA turbine-powered airliners, reporting not only the top-level aircraft performance parameters but also the relative changes in the masses and drag contributions of the individual aircraft components affected by the change in fuel type. This would allow for a deeper understanding of the reasons why the optimal tank integration strategy seems to depend on the aircraft range category. To achieve this objective, a hydrogen tank sizing and integration methodology is proposed and implemented in an existing aircraft design framework to consistently analyse how different combinations of fuselage diameter and tank structure, layout, shape, venting pressure and pressure control methods affect key aircraft level parameters, for different aircraft categories.

The structure of this paper is the following: Section 2 describes the chosen design and analysis framework functioning and capabilities, with a particular focus on the tank sizing. Section 3 presents the validation and compares the results from this research to those found in the literature. Section 4 presents the results in terms of performance of the $\text {L}\text {H}_\text {2}$ aircraft versions generated using this method and compares them to their kerosene counterparts designed with the same framework and Section 5 presents the conclusions of the research and offers inspiration for future work. This article is an extension of the thesis by Onorato [20].

## Methodology

A method to size liquid hydrogen aircraft must be defined to obtain the design framework necessary for this research. In addition, this method has to be integrated in an existing aircraft conceptual design framework to make this framework capable of sizing both liquid hydrogen and kerosene aircraft. This section first briefly describes the overall design process and its implementation, then dives into the $\text {L}\text {H}_\text {2}$ tank design and integration, and finally discusses some design choices in the estimation of the wing, propulsion system and landing gear masses.

### Aircraft design framework

The aircraft synthesis framework that has been modified and employed for this research is the Aircraft Design Initiator (or in short, Initiator). The Initiator is an in-house automated design synthesis tool, under continuous development at the Flight Performance and Propulsion section of the Faculty of Aerospace Engineering at Delft University of Technology. The software contains a design convergence loop over several disciplinary analyses, including handbook methods (a.o. [21–23]), empirical data and physics-based methods (a.o. ESDU). It was initially conceived as part of the European project Aerodesign (FP7) and has supported other European projects such as RECREATE (Horizon 2020), Smart Fixed Wing Aircraft (Clean Sky I) and Parsifal (Horizon 2020). Currently, it is being used in NOVAIR (Large Passenger Aircraft framework) and CHYLA (thematic topic) under Clean Sky 2.

The Initiator can be used to assess the impact of small and large changes to the aircraft on so-called key performance indicators (KPIs) in the conceptual design of CS-25 aircraft, supporting both propeller-powered and turbofan-powered aircraft, hybrid-electric aircraft (with distributed propulsion), as well as conventional tube-and-wing aircraft and (to some degree) blended-wing-body aircraft ([24]) and box-wing aircraft ([25]). A description of the Initiator can be found in Elmendorp et al. [26] and recent validation studies on various aircraft can be found in [27–29].

The Initiator relies on a convergence process for the synthesis, where the aircraft is iteratively updated until a predefined set of KPIs converges below a prescribed threshold. This is a process of design “feasilisation” [30], hence it does not perform an actual optimisation but rather synthesises a feasible design around the top-level requirements.

#### Existing aircraft sizing framework

**Fig. 1:**
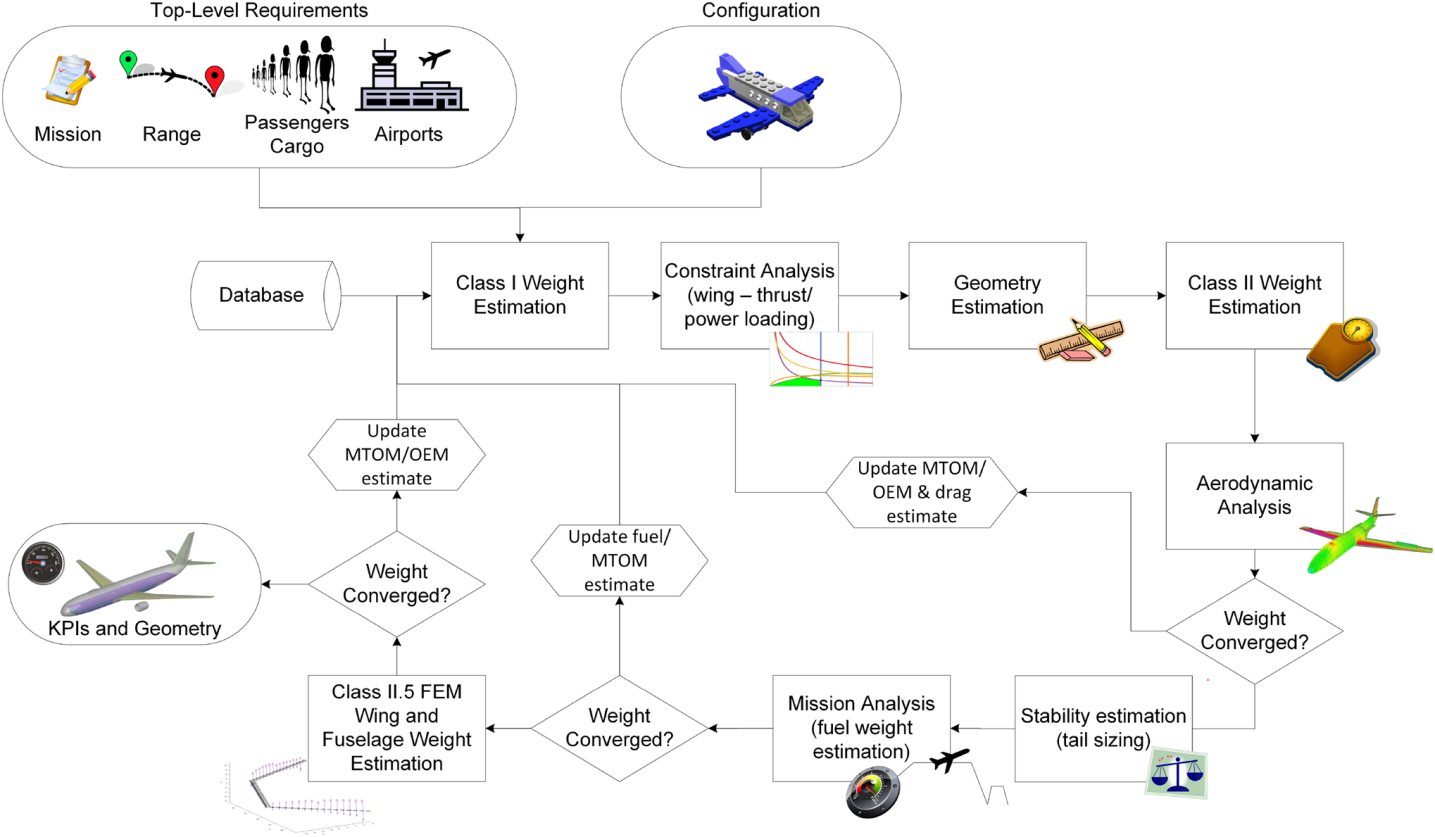
Flow chart of the aircraft design process

The Aircraft Design Initiator framework consists of a series of disciplinary analysis and sizing processes arranged into three main, partially nested, convergence loops (see Fig. 1). In the first convergence loop, reference aircraft data and the fuel-fraction method are combined to provide a Class 1 estimate of the maximum take-off mass. Subsequently, from the combination of a user-specified set of top-level aircraft requirements and performance requirements derived from regulations (FAR/CS-25), the required thrust (or power in case of propeller aircraft) and wing size are computed using a typical constraint analysis in a wing vs. thrust (or power) loading diagram. In the next step, the geometry of the aircraft is generated, following an inside-out aircraft design process using also empirical sizing rules and user-specified input on the aircraft configuration. Based on this geometry, the aircraft operational empty mass is estimated using the method from Torenbeek [21] and the aerodynamic properties are estimated using a vortex-lattice method complemented by a parasite drag estimation based on Torenbeek [21], Roskam [22], Obert [23] and ESDU methods. The system masses and the aircraft aerodynamic properties are fed back to the start of the loop until their differences with the previous iteration fall below a certain threshold.

In the second loop, the horizontal tail is no longer sized according to tail volume coefficients, but using an X-plot method [21] that uses requirements on stability and control and also re-positions the wing. A more accurate mission analysis [31], sensitive to changes in the centre of gravity and using the trimmed drag polar, replaces the fuel-fraction method. These more refined analyses impact the fuel weight and empty weight estimation, and consequently the results of all the previously completed disciplines.

In the third loop, the wing and the fuselage structures are sized using a combination of finite-element method and semi-empirical equations, to obtain more accurate, physics-based, masses, through methods by [26, 32–35], respectively. This includes typically sizing load cases for the wing structure at 2.5 G pull-up at maximum zero fuel weight, as well as a 2.5 G pull-up at maximum take-off weight.

Note that Fig. 1 only shows the process on an aggregate level; many analyses actually consist of smaller methods. For example, “Geometry Estimation” contains more than 20 individual modules determining the aircraft geometry. Dependencies between modules are automatically triggered, for example, when “Class-II Weight Estimation” is triggered, it first evaluates all the preceding modules, including the Class-I, if these have not been evaluated yet in the current iteration.

#### Overview of liquid hydrogen tank sizing process

**Fig. 2:**
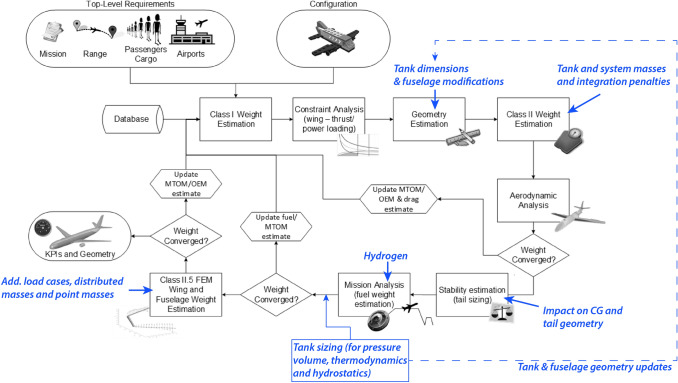
Required modifications to the aircraft design process to accommodate integration of $\text {L}\text {H}_\text {2}$ tanks

The usage of liquid hydrogen implies that large fuel tanks must be placed somewhere in the aircraft. The masses and dimensions of these tanks depend not only on structural and thermal tank design choices but also on the amount of fuel burned during the mission. Since the latter depends on the aircraft mass and drag, the necessity to use an iterative design process arises. Necessarily, the overall aircraft design process will also require some modifications. The traditional inside-out fuselage generation, where a 2D cabin section is first created around the required passenger/cargo dimensions and then extended in lengthwise direction to contain the required payload volume, works for conventional airliners where all (or often the majority) of the kerosene can be contained in wing tanks. However, since hydrogen has a low volumetric energy density, tank design and integration must be incorporated in the geometry estimation and in the conceptual design (handbook) methods. This causes extra difficulties for the design process that only allows to estimate the required fuel capacity after a mass estimation and mission analysis (requiring the mass estimate for the equations of motion) have been performed. Although a (modified) fuel-fraction method could be used to provide an initial estimate (as for kerosene), this does not consider the tank layout (which can have a significant influence). Hence, the fuselage geometry must be modified in such a way that it can handle an initial estimate as well as the more accurate results from a mission analysis (for a second or later iteration of the process), the tank must be sized to sustain the necessary loads, the fuel system must be modified and some changes to the airframe sizing are required (fuselage, wing, propulsion system and landing gear). The required modifications are highlighted on top of the aircraft design process in Fig. 2. The various parts of the modified aircraft sizing process for $\text {L}\text {H}_\text {2}$ aircraft will be presented in detail in Sects. 2.2, 2.3, 2.4 and 2.5, respectively.

### Fuselage adaptation to tank integration

In this research, two fuselage tank layout options have been analysed. The first option consists of placing a single, large-diameter tank aft of the passenger cabin. The second option consists of using a combination of two smaller tanks: a large-diameter one placed aft of the passenger cabin carrying most of the fuel, and a small-diameter one placed forward of the passenger cabin. The latter carries just enough fuel to retain the small c.g. range typical of kerosene aircraft and preserves the cabin-cockpit connection, by being shifted in lateral direction to create a 70 cm wide corridor.

**Fig. 3:**
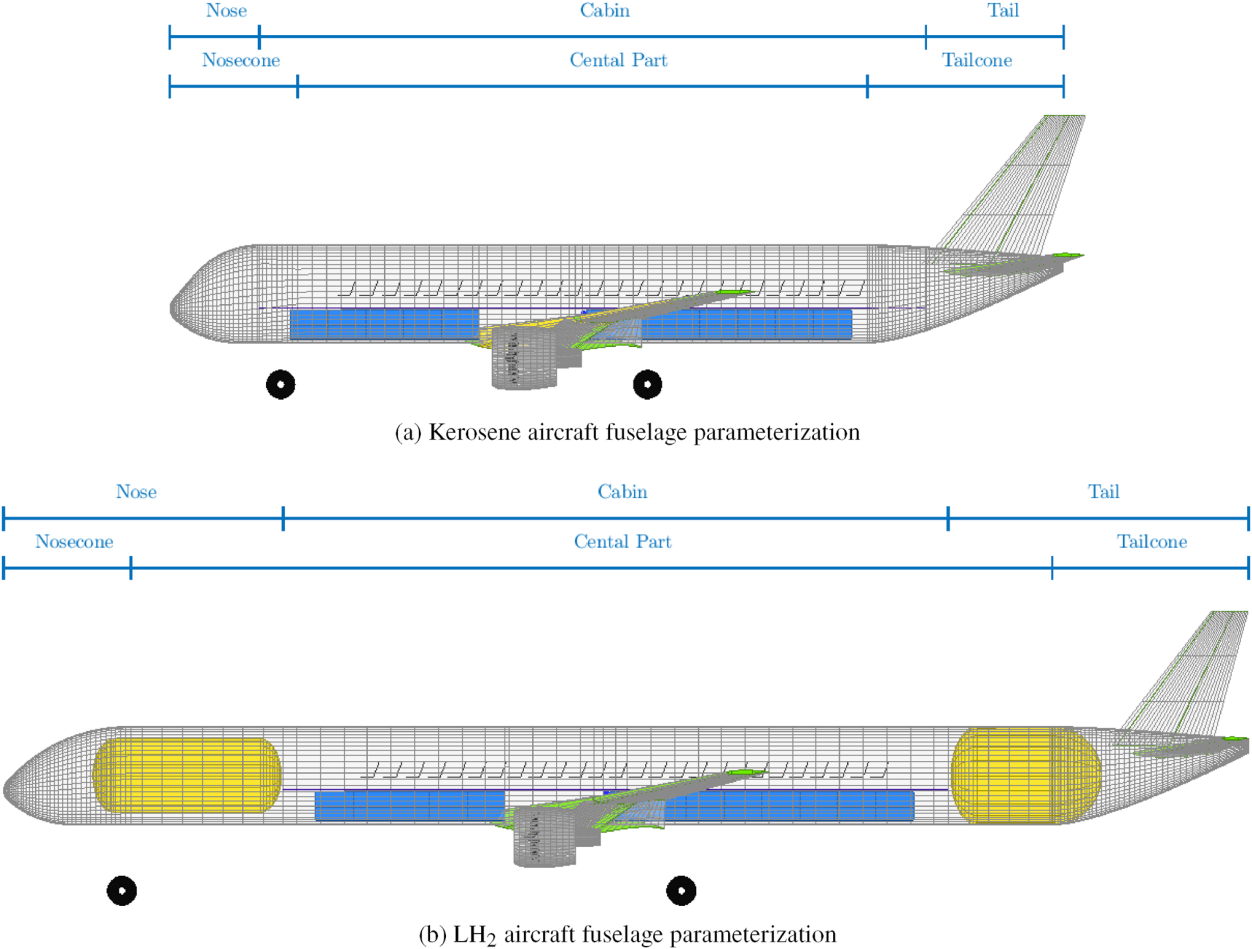
Fuselage sections and location of $\text {L}\text {H}_\text {2}$ tanks, fuel tanks in yellow

The fuselage is modelled in this method using three section compartments, namely the cabin, the nose and the tail and three section shapes, which are the central untapered part, the nose-cone and the tail-cone (see Fig. 3). While the nose-cone and the tail-cone section lengths remain proportional to the fuselage radius for both the kerosene and the $\text {L}\text {H}_\text {2}$ aircraft, the nose and the tail section lengths start as fractions of the fuselage radius and are extended in the $\text {L}\text {H}_\text {2}$ version to accommodate the fuel tanks. In case a forward tank is present, a nose section extension equal to the tank length is used, to preserve the cockpit space. In case an aft tank is present, the tail section extension is made such that the start of the tail-cone section coincides with the longitudinal coordinate of the start of the tank-aft end-cap. This way, the tank fit is ensured, the tanks most efficiently use the untapered fuselage section and sufficient volume in the tail is left for the allocation of the auxiliary power unit and the empennage structure.^14^

The fuselage structural mass is determined using a combination of finite-element method and semi-empirical equations described in Refs. [32–35]. The longitudinal and lateral fuselage loads experienced by the fuselage are analysed for several flight conditions and the most critical ones are used, in combination with the pressurisation loads, to size the skin, the stringers, the frames and the cabin floor for the cabin section. When the fuselage houses $\text {L}\text {H}_\text {2}$ tanks in the tail and/or nose section compartments, the fuselage sections containing them are also sized using this finite-element method. Note that when an aft tank is placed behind the aft pressure bulkhead, the fuselage skin is only sized to cope with the loads not associated to pressurisation, as the fuselage section containing the tank is not pressurised. Semi-empirical equations from Howe [36] are used to estimate the masses of the fuselage nose and tail shells, the passengers, cargo and crew floors, the forward and aft pressure bulkheads, the windows and the windshield, the cabin doors and the landing gear bays.

### Tanks sizing and integration

**Fig. 4:**
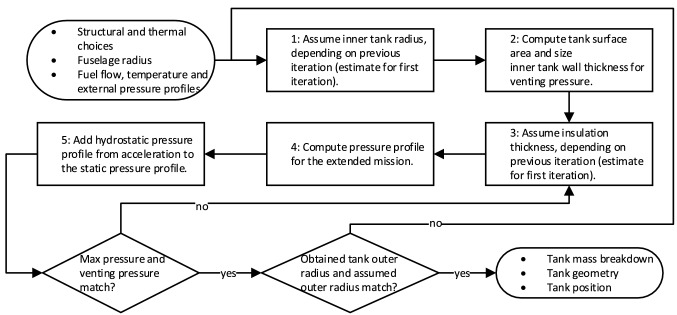
Sizing process for $\text {L}\text {H}_\text {2}$ tanks

$\text {L}\text {H}_\text {2}$ tank sizing is required after each mission analysis iteration (when an update of the fuel mass required is available). The output is the tank geometry and the tank mass, which includes the mass of the support system and the mass of the unusable fuel, and which must be used in the other sizing methods (as illustrated schematically in Fig. 2). The tank sizing combines several disciplines: the sizing of the structure to withstand pressurisation, the calculations of the thermodynamic and the hydrostatic loads and the determination of the fuel mass and the tank volume allowances. These will be covered in detail in Sects. 2.3.1 through 2.3.5. The tank sizing iterative process is described schematically in Fig. 4.

#### Design options

**Fig. 5:**
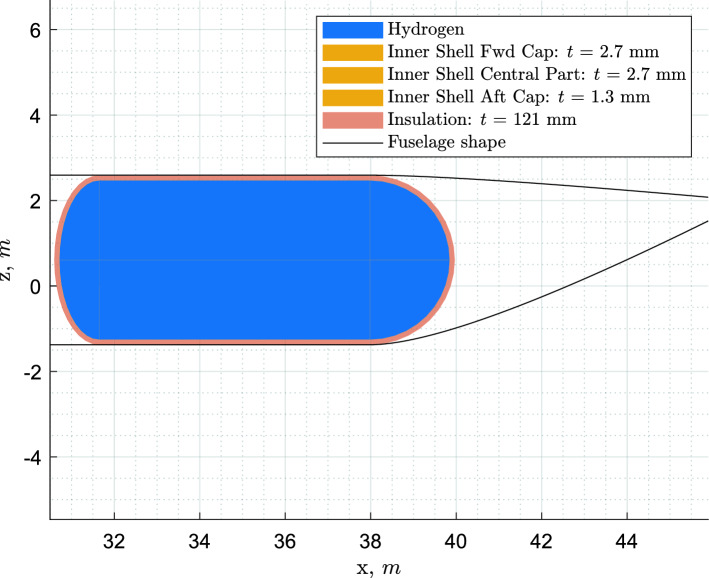
Example of integral tank cross-section generated by the method

**Fig. 6:**
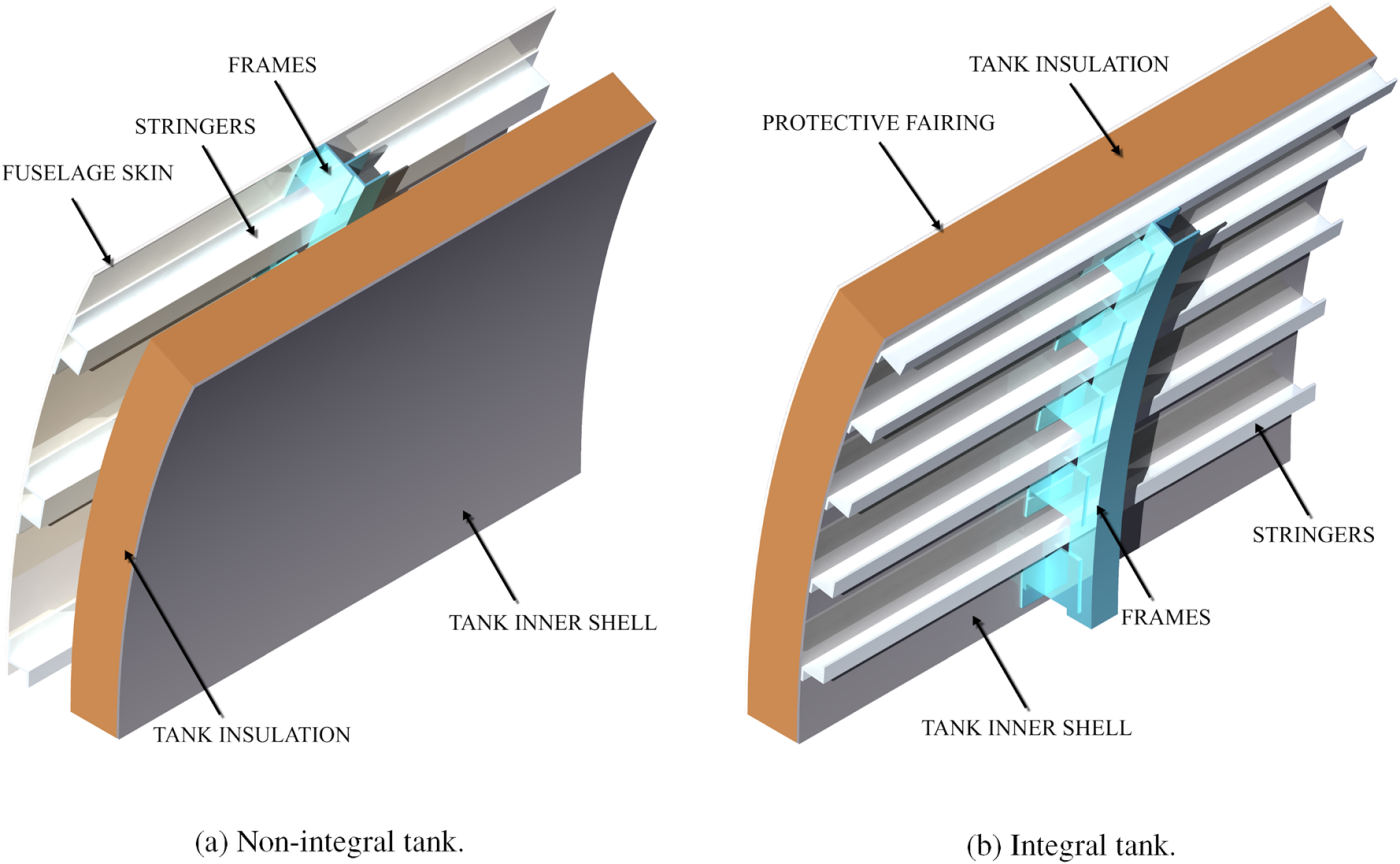
Artist impression of tank structural options. In the model, the stiffening elements are part of the fuselage structure

The tank is modelled as an inner structural shell externally covered by a uniform layer of insulating material (see Fig. 5). Concerning the tank position, the option to choose between a single aft tank and a combination of forward and aft tanks is available. Concerning the tank structure, the option to choose between integral and non-integral tank is available (see Fig. 6). For an aft, non-integral tank, the option to place it inside or outside of the pressurised fuselage region is also present. A forward tank is, instead, always non-integral and always placed in the pressurised fuselage region (at a pressure slightly lower than the pressure in the cabin, for safety reasons), because of its size and location. The fuselage diameter can be varied to reduce the length of the fuselage extension. The tank venting pressure and the use of direct gas venting are also among the design options.

#### Pressurisation loads

**Fig. 7:**
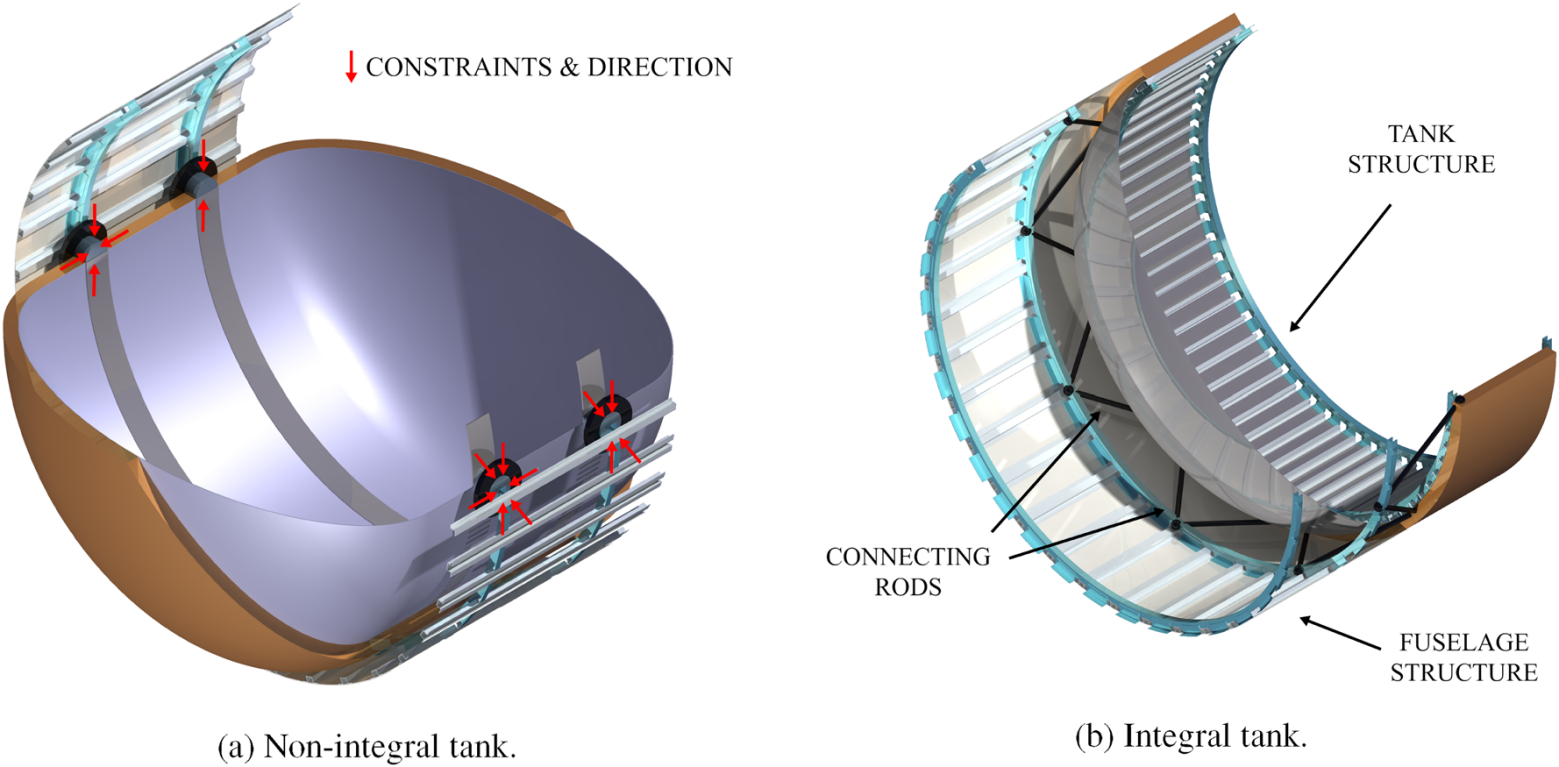
Artist impression of tank attachments options. In the model, the stiffening elements are part of the fuselage structure

If the non-integral tank option is selected, a four-point tank support system connects the tank to the fuselage (see Fig. 7a). This mounting solution, presented in Brewer’s book Hydrogen Aircraft Technology [17], allows for thermal contraction and expansion and prevents the fuselage deflections from affecting the tank. For this tank option, the outer tank radius is set equal to 93.8% of the external fuselage radius (less in the case of a forward tank) to provide space for the fuselage structure and system routing. This value represents the cabin radius to the outer fuselage radius of the A320-200.

In case the integral tank option is selected, a stiffened tank structure replaces the fuselage section at the tank location. Truss structures with low thermal conductivity connect the tank to the fuselage section forward and aft of it (see Fig. 7b). A protective fairing would be placed on top of the insulation and equipment and system routing tunnels would be added on top of it. For this tank option, the outer tank radius is set equal to the fuselage external radius. The stiffening elements for the integral tank (stringers, frames and skin—not having to cope with pressurisation) and the truss structure for the integral tank are not directly sized, but their mass is assumed to be equal to the mass of the stringers, frames and respective skin thickness already included in the fuselage structural sizing, housing the tank structure. When aluminium is used for both the fuselage and the tank structure (as done in this paper), the above assumption is conservative since the tensile yield strength, the ultimate yield strength, the fracture toughness, and the yield modulus increase at cryogenic temperatures for the adopted Aluminium 2219-T8 series [37].

For both tank options, the central part of the structural shell, which resists the pressure load only, is sized using the following equation (Barlow’s formula):

1 $$\begin{aligned} t_{\text {shell}_\text {central}} = \frac{(P_\text {vent}-P_\text {out})\cdot r_\text {shell}}{\sigma \cdot e_{w}}. \end{aligned}$$

According to Eq. 1 the structural/inner shell thickness ($t_\text {shell}$) is equal to the difference between the venting pressure ($P_\text {vent}$) and the air pressure outside of the tank ($P_\text {out}$), multiplied by the tank structural shell radius ($r_\text {shell}$), divided by the allowable stress of the inner shell material ($\sigma$) and a safety factor ($e_w$) equal to 0.8. For the non-integral tank, $P_\text {out}$ is set equal to the cabin pressure at the minimum cabin altitude of 2000 *m* ($P_{\text {cabin}_\text {min}}$), whereas for the integral tank, it is set equal to the atmospheric pressure at the maximum flight altitude encountered in the flight profile ($P_{\text {amb}_\text {min}}$). Values of 172 *MPa* ($\sigma _{a,R_1}$) and 234 *MPa* ($\sigma _{b}$) were obtained by Brewer, respectively, for the operating design stress and the ultimate design stress, for the 2219-T851 aluminium alloy ($\rho _\text {al} = 2840 \text {kg/m}^3$) at -252$^{\circ }$C, for 40,000 cycles, a stress ratio (minimum stress to maximum stress values) of 0.43 ($R_1$) and with a fatigue quality index of 5 [17]. The stress ratio experienced by the inner shell of the tanks designed in this research ($R_2$) is not a fixed value, as the maximum tank pressure (venting pressure) is a design choice and the air pressure outside the tank depends on the tank structural choice and on the aircraft cruise altitude (in case of integral tank structure). For this reason, $\sigma$ is obtained by applying the Goodman relation twice, first to obtain the fatigue limit for completely reversed loading (see Eq. 2) and then to obtain the fatigue limit for the tank inner shell stress ratio (see Eq. 3):

2 $$\begin{aligned}&\sigma _{a,-1} = \frac{\sigma _{a,R_1}}{1-\frac{\sigma _{a,R_1}\cdot 0.5\cdot (1+R_1)}{\sigma _b}}, \end{aligned}$$

3 $$\begin{aligned}&\sigma = \sigma _{a,R_2} = \frac{\sigma _{a,-1}}{1+\frac{\sigma _{a,-1}\cdot 0.5\cdot (1+R_2)}{\sigma _b}}. \end{aligned}$$

For the ellipsoidal end-caps with a 2:1 major-to-minor axis ratio, the thickness obtained for the central section is directly used, while for ellipsoidal end-caps with a 1:1 major-to-minor axis ratio (hemispheres) half of the thickness obtained for the central section is used, because of the halved circumferential stresses. The hemispherical caps, which are lighter but longer, were adopted for the aft cap of the aft tank, as they did not lead to an increase in fuselage length (see the explanation of the aft tank placement in Sect. 2.2).

It is worth mentioning that a sensitivity analysis on $\sigma$ showed that a 25% reduction in $\sigma$ would entail, for an SMR aircraft, a 17% increase in tank mass, 1.3% increase in OEM, 0.9% increase in MTOM and 0.4% increase in specific energy consumption per passenger per km (SEC).

For the non-integral tank option, the mass of a tank support system is set equal to 1.8% of the tank mass (including fuel). The 1.8% is the value found by Brewer [17] for this type of support system for its case-study aircraft, and the assumption is made that the tank support system mass is linearly proportional to the mass it supports. In addition, depending on the number of engines and tank layout, the masses of the tank dividers are added to comply with the FAA requirement of having each engine supplied by a different tank during take-off (this solution is proposed by Brewer [17]). The mass of this tank divider is set equal to the average between the forward and the aft cap inner shell masses, as it would have a similar structure.

#### Thermodynamic loads

The hydrogen boil-off due to heat entering the tank together with its consumption in the engines causes continuous pressure variations inside the tank. Lin et al. [38] investigated methods of pressure control for ${\hbox {LH}_2}$ tanks using a homogeneous thermodynamic model, with liquid and vapour phases at a uniform temperature equal to the saturation temperature of the cryogenic fluid at the total tank pressure. Among their proposed pressure control systems, the one including fluid mixing and direct venting was selected. The pressure fluctuation for this system is expressed by the following equation:

4 $$\begin{aligned}&\frac{dP}{dt}=\frac{\phi }{V}\cdot \left[ {\dot{Q}_w}+\dot{W}_\text {mix}-\dot{m}_{g}\cdot h_{lg} \right. \nonumber \\&\quad \left. \cdot \left( 1+\frac{\rho _g}{\rho _l-\rho _g}\right) -\dot{m}_{l}\cdot h_{lg}\cdot \left( \frac{\rho _g}{\rho _l-\rho _g}\right) \right], \end{aligned}$$

where the pressure change rate $$\left( \frac{dP}{dt}\right)$$ is equal to the ratio between the energy derivative of hydrogen ($$\phi$$) and the tank fluid volume (*V*), multiplied by the summation of four terms. The first term is the tank heating rate ($$\dot{Q}_w$$) and the way it is obtained is explained later in this section. The second term is the rate of work done on the fluid ($$\dot{W}_\text {mix}$$). The fluid mixing is used to destroy the fluid temperature stratification and to induce condensation at the liquid–vapour interface, resulting in a reduction of the tank pressure. The mixer power required to circulate the tank fluid adds to the system a certain amount of energy which eventually becomes heat and increases the net fuel energy [38]. The third term is the venting of the gaseous phase ($$\text {G}\text {H}_\text {2}$$), equal to the mass flow rate of the gaseous phase ($$\dot{m}_{g}$$), times the latent heat of vaporisation ($$h_{lg}$$), times 1 plus the ratio between the density of the gaseous phase ($$\rho _g$$) and the difference between the density of the liquid phase ($$\rho _l$$) and the density of the gaseous phase. The fourth term is the venting of the liquid phase, equal to the mass flow rate of the liquid phase ($$\dot{m}_{l}$$), times the latent heat of vaporisation, times the ratio between the density of the gaseous phase and the difference between the density of the liquid phase and the density of the gaseous phase. The energy derivative $$\phi$$, which represents the pressure rise per energy input per volume (Pa/(J / m$$^3)$$), is computed as follows:

5 $$\begin{aligned} \phi =\left( \rho _\text {mean}\cdot \left( \frac{\partial u}{\partial P}\right) _{\rho _\text {mean}}\right) ^{-1}. \end{aligned}$$

**Fig. 8:**
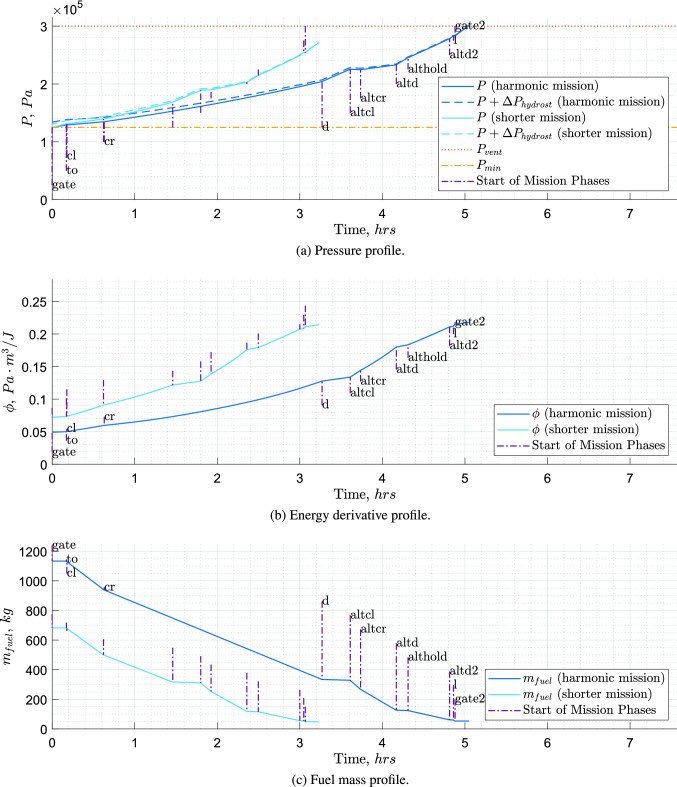
Example of pressure, energy derivative and fuel mass profiles of a tank, both in case of harmonic/maximum fuel mission and in case of shorter mission. Flight phases: gate (disconnect from boil-off recovery adapter), to (start of take-off), cl (start of climb), d (start of descent), altcl (start of alternative climb), altcr (start of alternative cruise), altd (start of alternative descent), althold (start of hold), altd2, start of second alternative descent), l (start of landing), gate2 (arrival to gate, until reconnection to boil-off recovery adapter)

According to Eq. 5, the energy derivative is equal to the reciprocal of the product between the fuel mean density ($\rho _\text {mean}$) and the partial derivative of the specific internal energy (*u*) to the tank pressure. For this research, the venting of fluid and gas has been separated into two components to highlight that they represent different functions. The venting of the liquid phase represents the fuel drawn by the tank to feed the engines, whereas the venting of the gaseous phase represents direct venting to the exterior of the aircraft system, with the sole purpose of lowering the tank pressure. Equation 4 is integrated with a time step of 10 *s* from the moment the aircraft is disconnected from the refuelling station and the boil-off recovery adapter to the moment the aircraft has landed and is reconnected to the boil-off recovery adapter (see Fig. 8a). The starting pressure is set equal to $P_{\min }$ and measures 125 *kPa*. This pressure is sufficient to prevent air from entering the tank, with some safety margin [17]. Figure 8b and c shows the energy derivative profile and fuel mass profile, respectively, for the pressure profile in Fig. 8a. As indicated by Brewer [17], through the boil-off recovery adapter, the aircraft operator can return gaseous boil-off to ground facilities for re-liquefaction, during refuelling or prolonged periods at the gate with full tanks.

The mission for which the tank is being sized is the harmonic (maximum range with maximum payload) plus diversion, but logically (tank sized by fuel for this mission), this also becomes the maximum fuel mission (maximum range at MTOM and full tanks). How this compares to kerosene aircraft in terms of payload-range diagram can be visualised in Fig. 10.

When the tank designed for the harmonic mission is used for a shorter mission, the values for $\rho _\text {mean}$ are lower, and as a consequence, the values for $\phi$ are higher (see Fig. 8b). The values of $\dot{m}_{l}$ are also slightly lower, due to the lower fuel mass carried. Despite the increase in pressurisation rate due to the higher $\phi$ values and the lower $\dot{m}_{l}$ values, the shorter mission time over which Eq. 4 is integrated was found to be the dominant factor in determining the maximum reached pressure. Due to the shorter mission time, these shorter missions are non-critical (see Fig. 8a). When this tank is used for a mission as long as the harmonic one but with a lighter payload, the values for $\rho _\text {mean}$ and $\dot{m}_{l}$ are also slightly lower due to the fuel masses and, as a consequence, the pressurisation rate is slightly higher. For those missions some extra fuel has to be carried and vented. This extra fuel would have a small impact in terms of both added mass and energy consumption, as the amount required would be small and venting would occur mainly, if not entirely, during the diversion phases.

**Fig. 9:**
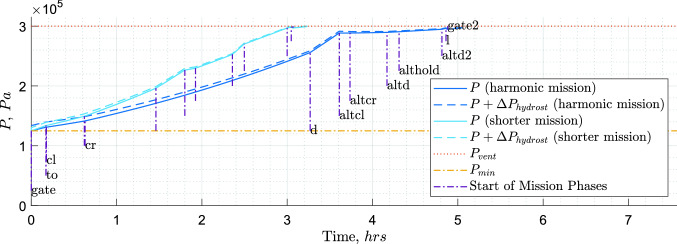
Tank pressure profile for one of the designed aircraft. Fuel direct venting starts at the beginning of the alternative cruise for the harmonic mission and at the beginning of the hold for the shorter mission. Flight phases: see Fig. 8a

If direct gas venting is used, the most efficient moment to vent is the end of the mission, where $\phi$ has the largest values (see Fig. 8b), as the pressure drop due to venting is proportional to $\phi$ (see Eq. 4). Moreover, it could be argued that by designing a tank that reaches venting pressure just after the regular mission time and starts venting gaseous hydrogen during the diversion phases, a lighter tank could be designed without the drawback of a larger effective energy consumption due to vented hydrogen. This is the case because for a regular mission no venting would be required and the excess hydrogen carried for the venting, necessary in the eventuality of a mission extension, could be recovered on ground. The pressure profile for this type of mission is presented in Fig. 9.

The heat flow through the tank is computed by considering only conduction through a single insulation layer with uniform thermal properties. Indeed, the hydrogen itself, being kept as a homogeneous liquid–gaseous fuel mixture at the saturation temperature, does not offer any thermal resistance, hence the inner tank surface can be equalled to the fuel temperature of 20 *K* ($T_{\text {fuel}}$). At the same time, by neglecting the radiative heat balance at the tank surface, the outer tank surface temperature can be equalled to the air temperature outside of the tank ($T_{\text {out}}$). $T_{out}$ is set equal to the cabin temperature of 296 *K* ($T_{\text {cabin}}$) throughout the whole integration period for the non-integral tanks, as these tanks are placed within the pressurised fuselage section. For the integral tank, instead, $T_{\text {out}}$ follows the ambient temperature corresponding to the flight altitude profile, in a hot day scenario^15^ ($T_{\text {amb}}$, ISA plus 24.5 *K*). The simple solution to the heat conduction through flat plate (Eq. 6) is used because the insulation layer thickness ($t_\text {ins}$) is an order of magnitude smaller than the tank radius:

6 $$\begin{aligned} \dot{Q}_w = \frac{(T_\text {out}-T_\text {fuel})\cdot k_\text {ins}\cdot A}{t_\text {ins}}. \end{aligned}$$

According to Eq. 6, the tank heating rate is equal to the difference between the air temperature outside of the tank and the fuel temperature, times the effective thermal conductivity of the insulation material ($k_\text {ins}$), times the outer tank surface (*A*), divided by the insulation layer thickness. The value of $k_\text {ins}$ at $T = (T_\text {amb}+T_\text {fuel})/2$ was selected to approximate the overall performance of the insulation layer. $k_\text {ins}$ of polyurethane foam ($\rho _\text {ins}=32\,\text {kg/m}^3$) at 170 *K* is 0.022 $\text {W/(mK)}$ [17]. The technology readiness level of foam insulation is higher than the one for vacuum insulation and there are safety concerns regarding vacuum insulation in the case of a loss of vacuum. For the current requirement of holding time, foam was sufficient and the impact of the choice of insulation on the overall system-level was limited.

Worth mentioning is that a sensitivity analysis on $k_\text {ins}$ showed that a 25% increase in $k_\text {ins}$ would entail, for an SMR aircraft, an 8% increase in tank mass, 5% increase in tank length, 1.4% increase in OEM, 1% increase in MTOM and 0.5% increase in SEC. $\dot{Q}_{w}$ is increased by 30% to account for the extra heat leaking through the support structure and the piping, as suggested by Verstraete [39].

#### Hydrostatic loads

Besides the increase in pressure due to hydrogen evaporation, the tank also experiences hydrostatic pressure increments from aircraft accelerations. These pressure increments depend linearly on the magnitude and direction of the aircraft accelerations and the tank dimensions. A simple model to estimate these pressure increments, taken from the CS-25 regulations and also used for $\text {L}\text {H}_\text {2}$ aircraft by Gomez and Smith [40], is expressed by the following equation:

7 $$\begin{aligned} \Delta P_{hydrost}=\rho _\text {mean} \cdot K \cdot g \cdot l \end{aligned}$$

According to Eq. 7, the hydrostatic pressure increment from aircraft accelerations ($\Delta P_{\text {hydrost}}$) is equal to the fuel mean density, times a coefficient for the linear acceleration (*K*), times the gravitational acceleration (*g*), times the characteristic length in the direction of the acceleration (*l*). Note that $\rho _\text {mean}$ is computed at every integration step, for *K* the critical value of 9 for forward acceleration is used and $l_\text {tank}$ is used for *l*.

#### Extra allowances

The mass of fuel required in the tank the moment the aircraft is disconnected from the refuelling station is the sum of the fuel burned during the extended mission, plus the mass of $\text {G}\text {H}_\text {2}$ vented during flight (if any), plus a 0.3% trapped fuel allowance [17], plus a pressurisation fuel allowance. The pressurisation fuel allowance accounts for the mass of $\text {G}\text {H}_\text {2}$ present in the tank at the end of the diversion (which is about 4.3% of the burned mass at $P_\text {vent}= 250\,kPa$, which is a typical venting pressure value [15]). From a volume perspective, these unusable fuel components are added to the fuel volume. From a mass perspective, these components are added to the tank mass. From an energy perspective, except for the $\text {G}\text {H}_\text {2}$ vented during flight, these components are not accounted for because they can be recovered when the aircraft is on the ground. The internal volume of the tank is computed by adding to the fuel volume a 0.9% tank contraction–expansion allowance, a 2% ullage (i.e. non-fillable part) allowance and a 0.6% internal equipment allowance. The values of these allowances are retrieved from Brewer [17], except for the pressurisation fuel allowance, which is dependent on the choice of venting pressure.

### Fuel system sizing

The fuel system mass uses a modified version of the Class 2 Torenbeek estimation (Eq. 8 here, Table 8.9 in Torenbeek [41]):

8 $$\begin{aligned} m_\text {fuelSys}= 36.3\cdot (N_e+N_{ft}-1)+4.366\cdot N_{ft}^{0.5}\cdot V_{ft}^{0.333}. \end{aligned}$$

According to Eq. 8, the fuel system mass (excluding $\text {L}\text {H}_\text {2}$ tank) ($m_\text {fuelSys}$) is equal to the summation of two terms. The first term is equal to 36.3 times the sum of the number of engines ($N_e$) and the number of fuel tanks ($N_{ft}$) minus 1. The second term is equal to 4.366 times the number of fuel tanks to the power of 0.5, times the fuel volume in litres ($V_{ft}$) to the power of 0.333.

The fuel system for a tube-and-wing turbine $\text {L}\text {H}_\text {2}$ airliner has been meticulously designed and described by Brewer [17]. This fuel system consists of a vent system, insulated fuel supply lines to the engines, heat exchangers to transfer the engine and the airframe heat to the cryogenic fuel, fuel quantity gauging equipment, refuelling and de-fuelling systems, fuel jettison system, pumps, valves, seals etc. When applying Eq. 8 to the $\text {L}\text {H}_\text {2}$ aircraft designed by Brewer, the value obtained for the fuel system is a fraction ($k_{fs}=0.50$) of the one by him reported. It is believed that using Eq. 8 and dividing the results by $k_{fs}$ a reasonable estimate for the $\text {L}\text {H}_\text {2}$ fuel system can be obtained for the aircraft under investigation in this research. This modified equation was applied to the case study from Silberhorn et al. (rear tank option) [19] and a mass of 744 kg, similar to the 781 kg indicated by the study, was obtained. From a centre-of-gravity perspective, the fuel system is placed at the centre of gravity of the tank.

### Wing, propulsion and landing gear

The wing mass is sized using the class II.V method developed by Elmendorp and La Rocca [24]. This method uses a finite-element beam discretisation approach to determine the internal forces and moments due to the critical load cases and to identify the minimum amount of primary structure required to withstand them. From the “ideal” primary structure (the wing-box) mass, the method described by Torenbeek [42] is used to correct the ideal primary structure mass (sheet taper, joints in skin-stringer panels, large cut-outs, etc.) and to estimate the mass of the secondary structure (leading and trailing edge, high-lift devices, ailerons, etc.)[. The wing structure and significant wing-mounted components, such as the fuel and the engines are, when present, included in the inertial loads. This makes the method suitable for application in this research as the absence of the fuel bending relief in the $\text {L}\text {H}_\text {2}$ aircraft is accounted for.

The propulsion system is also affected by the change in fuel type. The change in specific fuel consumption has been scaled with the change in fuel calorific value (x2.8). A thermodynamic cycle analysis carried out with an in-house code showed a $\pm 1\%$ difference in SEC, in design condition and with the engine optimised for the fuel type. Note that heat recuperation and intercooling options are not considered. The mass estimation methods used remain the ones implemented for kerosene aircraft, since the change in mass and volume of the system is known to be negligible [17].

The landing gear mass is estimated using Raymer’s method [43], because this method makes the sizing dependent on the aircraft landing mass and the landing gear height, two parameters which will sensibly differ between the kerosene and the $\text {L}\text {H}_\text {2}$ aircraft versions.

## Validation

Due to the absence of currently operational or even experimental $\text {L}\text {H}_\text {2}$ airliners, a validation of the complete tool using experimental data is impossible. Nevertheless, this section will provide insight into the suitability of the tool for obtaining meaningful data. Section 3.3 complements this validation section by comparing the aircraft designed in this study to counterparts in literature.

### Validation of aircraft design software for kerosene aircraft

The kerosene version of the aircraft design software, which serves as the backbone for the automated aircraft design process, has been validated in Elmendorp et al. [26, 44] and since then it has been continuously improved and expanded. It was adopted in several recent works, and additions and improvements were validated in Brown and Vos [29], Vos and Hoogreef [25] and Hoogreef et al. [27, 28]. For this study, a validation is performed for three aircraft representing the regional (REG), short/medium-range (SMR) and large passenger aircraft (LPA) aircraft categories, namely the ATR72-600, the A320neo and the A330-300. The version of these aircraft generated using the method described in this paper will be referred to with the acronyms referring to their category, followed by “-JA1”—for Jet A-1. These aircraft are also the ones which will be investigated in Sect. 4. The main mission requirements and performance/configuration parameters are presented in Table 1.

**Table 1:** Aircraft main mission requirements and performance parameters for the validated (and investigated) aircraft. The references aircraft are the ATR72-600, A320neo (weight version WV0055), and A330-300 (weight version WV082 (c))

Parameters	ATR72-600$^{\mathrm{a}}$ (REG-JA1)	A320neo$^{\mathrm{b}}$ (SMR-JA1)	A330-300$^{\mathrm{b}}$ (LPA-JA1)
Number of passengers	72 (1 class)	150 (2 classes)	295 (3 classes)
PLM (*t*)	7.50	19.30	45.60
$M_\text {cruise}$	0.44	0.78	0.82
$h_\text {cruise}$ (m)	5200	11,278	11,887
Harmonic range (km)	926	4560	7674
Take-off distance (m)	1333	2180	2900
Approach speed (m/s)	58.1	67.7	70.5
Airworthiness Reg	FAR-25	FAR-25	FAR-25
Loiter time (min)	30	30	30
Diversion range (km)	160	370	370
$C_{L_\text {max}}$ landing	2.70	2.95	2.54
$C_{L_\text {max}}$ take-off	2.40	2.45	2.14
Wing aspect ratio	12	10.5	10.1
BSFC (g/(kWh))	400	–	–
	(142.9 for $$\text {L}\text {H}_\text {2}$$)		
TSFC (*kg*/(*Ns*))	–	1.443*E* – 5 [45]	1.689*E* – 5
		(0.5154*E* – 5 for $\text {L}\text {H}_\text {2}$)	(0.6030 for $\text {L}\text {H}_\text {2}$)

^a^Data from Jane’s “All the world’s aircraft” and ATR 72-600 Fact sheet https://www.atr-aircraft.com/wp-content/uploads/2020/07/Factsheets_-_ATR_72-600.pdf - visited: 29 April 2022

^b^Data from Jane’s “All the world’s aircraft” and Airbus Aircraft characteristics airport and maintenance planning. https://www.airbus.com/en/airport-operations-and-technical-data/aircraft-characteristics - visited: 29 April 2022

**Fig. 10:**
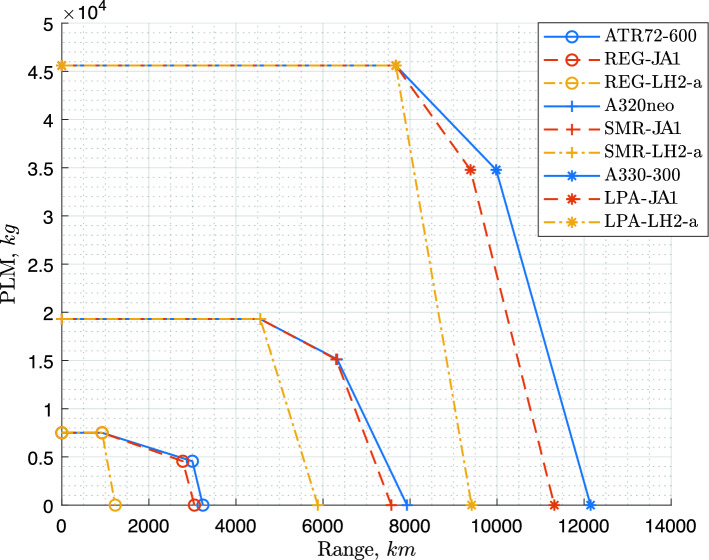
Payload-range diagrams of the reference aircraft and of the kerosene and $\text {L}\text {H}_\text {2}$ versions designed using the Initiator

Table 2 presents the output of the validation. Note that the FEM-based mass estimations for wing and fuselage have been tuned per aircraft category, to achieve the mass fractions of the wing and the fuselage which are typical of the reference aircraft chosen [21, 23]. This tuning is later applied to the hydrogen versions of these categories. This ensures that the right importance is given to these two large structural mass components. It can be seen that the results are well within the expected accuracy for the conceptual design methods that have been applied, giving confidence in the framework for modification for hydrogen aircraft design. In the results section of this article, a payload-range diagram is presented for the reference aircraft and their hydrogen and kerosene counterparts designed in this article. This diagram, presented in Fig. 10, shows similar performance for the kerosene versions except for slightly worse off-design performance. (The design point used in this article is the harmonic point, i.e. the first kink).

**Table 2:** Comparison table for the validated (and investigated) aircraft: ATR72-600 $^{\mathrm{a}}$, A320neo (WV0055)$^{\mathrm{b}}$ , A330-300 (WV082(c))$^{\mathrm{b}}$

Parameters	ATR72-600	REG-JA1	$\Delta$ (%)	A320neo	SMR-JA1	$\Delta$ (%)	A330-300	LPA-JA1	$\Delta$ (%)
MTOM (*t*)	23.0	22.8	– 1	79.0	79.1	0	242.0	241.5	0
MZFM (*t*)	21.0	20.9	0	64.3	64.0	0	175.0	174.1	– 1
FM (harmonic) (*t*)	2.0	2.1	+5	14.7	15.1	+3	67.0	67.4	+1
OEM (*t*)	13.5	13.2	–2	45.0	44.8	0	129.4	128.6	– 1
*W*/*S* (N/m$^2$)	3698	3782	+2	6329	6347	0	6563	6545	0
*T*/*W*	–	–	–	0.3124	0.3101	– 1	0.2596	0.2660	– 2
*W*/*P* (N/W)	0.0611	0.0613	0	–	–	–	–	–	–
*S* (m$^2$)	61.0	59.1	– 3	122.6	122.3	0	361.6	361.8	0
*b* (m)	27.0	26.6	– 1	35.8	35.8	0	60.3	60.6	0
$l_\text {fus}$ (m)	27.2	25.3	– 7	37.57	36.1	– 4	62.67	59.8	– 5
$r_\text {fus}$ (m)	2.87	2.78	– 3	4.14	3.98	– 4	5.64	5.86	4
$S_\text {ht}/S$	0.19	0.19	0	0.25	0.26	+4	0.19	0.19	0

### Comments regarding the liquid hydrogen tank sizing method

Concerning the validation of the newly added $\text {L}\text {H}_\text {2}$ tank sizing method and the modification made to the fuel system, wing, and fuselage sizing methods, the following points are made:

- Concerning the tank sizing discipline, the point is made that only proven structural (Barlow’s equation, allowances and correlation data from Brewer [17], and hydrostatic pressure increment from FAR/CS-25 regulations) and thermodynamic models (model from Lin et al. [38], heat conduction through a flat plate) have been combined, using conservative assumptions.
- Concerning the fuel system sizing method, the semi-empirical Torenbeek mass estimation [41] was multiplied with a correction factor extracted from Brewer [17]. This modified equation was applied to the case study from Silberhorn et al. (rear tank option) [19], which indicated a mass of 781 kg, and a mass of 762 kg was obtained.
- Concerning the wing sizing method, the method developed and validated by Elmendorp and La Rocca [24] for kerosene aircraft is considered applicable, having verified its ability to account for the presence (or the lack, in case of $\text {L}\text {H}_\text {2}$ aircraft) of the fuel bending relief effect on the wing primary structure.
- Concerning the fuselage sizing, the method described in Hoogreef and Vos [32] for blended-wing-body cabins and generalised for tube-and-wing kerosene aircraft ([34, 35]) is considered suitable for $\text {L}\text {H}_\text {2}$ aircraft too, as it places the fuel and the fuel tank masses at their respective fuselage locations for the inertial load calculations.

### Comparison with the literature

To complement the validation, in absence of experimental data, it is decided to compare aircraft and tank configurations designed in this study with their counterparts found in literature, both in terms of the aircraft level performance and tank level performance. The aircraft identifiers in this section refer to the ones discussed in more detail in Sect. 4.

#### Aircraft level performances

The relative values of the main aircraft performance parameters with respect to their own kerosene baselines are reported in Table 3.

In the regional aircraft category (REG), the **REG-LH2-a** is compared to the small regional aircraft from the Cryoplane Project$^{9}$. Both studies estimate an increase in OEM, a negligible change in MTOM and an increase in SEC, with the aircraft from this study performing a bit better.

For the SMR category, the **SMR-LH2-b** is compared to the A320-like $\text {L}\text {H}_\text {2}$ aircraft with aft tank layout designed by Silberhorn et al. [19]. The assumption is made that the Silberhorn et al. used an integral tank, as although they did not specify, it would seem to be the logical choice (better performance). Both studies estimate an increase in OEM, a reduction in MTOM and an increase in SEC. Similar are also the increases in fuselage length and mass.

In the LPA category, the comparison shows more differences between studies. The **LPA-LH2-c** is compared to the LPA from McKinsey & Company $^{2}$ and the one from the Cryoplane Project $^{9}$. With respect to the McKinsey & Company study, the results from this research appear extremely optimistic. Nevertheless, the extremely high (1.63) tank gravimetric index assumed by McKinsey & Company is out of line with the rest of the literature, and potentially the cause of the inferior performances of their aircraft. The results from present article align better with the ones from the Cryoplane Project in terms of MTOM. The cause of the large difference in OEM variation is not understood, but it is not exactly known what the kerosene baselines were in those studies, and as can bee seen in Table 7 for the results of these large aircraft, the performance significantly depends on the fuselage diameter choice.

**Table 3:** Comparison of aircraft level performance for aircraft designed in this research and REG aircraft from the Cryoplane Project$^{9}$, SMR aircraft designed in this research and from Silberhorn et al. [19] and LPA from McKinsey & Company $^{2}$ and LPA from Cryoplane Project$^{9}$. Performance values relative to the kerosene baselines of the respective studies. Note that some values were not reported in those studies

Parameters	REG		SMR		LPA		
	REG-LH2-a	Cryoplane	SMR-LH2-b	Silberhorn	LPA-LH2-c	McKinsey	Cryoplane
$l_\text {fus}$	–	–	+27%	+22%	–	–	–
$m_\text {fus}$	–	–	+25%	+28%	–	–	–
$L/D_\text {mid-cruise}$	–	–	– 5%	– 5%	–	–	–
OEM	+12%	+17%	+11%	+11%	+8	–	+25%
MTOM	+1%	+0%	– 6%	– 9%	– 14%	+23%	– 15%
SEC	+6%	+14%	+6%	+7%	– 4%	+42%	+9%

#### Tank level performance

In addition to the aircraft level comparison, an analysis is made for the fuel masses and gravimetric indexes. These are reported and compared in Table 4. Overall, the results are similar to those found in literature and consistent over the different classes of aircraft giving confidence in the method. The differences with literature can be explained by the different assumptions. The results are summarised as follows.

In the regional category, the tank belonging to the **REG-LH2-a** is compared to the polyurethane tank of the REG aircraft from Verstraete et al. [15]. The two tanks in question carry amounts of fuel of the same order of magnitude and have similar gravimetric indexes. Note that Verstraete et al. used 172 *MPa* for yield strength ($\sigma _{a,R_1}$), despite lower stiffness ratio ($R_1$) values. They also use a much lower venting pressure, but similar insulation thickness, which may explain the difference in gravimetric index.

For SMR aircraft, the tank belonging to the **SMR-LH2-b** is compared to the aft tank of the SMR aircraft from Silberhorn et al. [19]. Both tanks have a similar maximum fuel mass and a similar gravimetric index. Furthermore, it can be noticed that the masses of the fuel systems (excluding tanks) are very similar.

For the largest aircraft, in the LPA category, the tanks belonging to the **LPA-LH2-c** are compared to the tanks of the single-deck aircraft from Verstraete et al. [15]. The two tanks in question, carry very different amounts of fuel, though in the same order of magnitude. Relatively similar tank gravimetric indices are found, which are likely impacted by the different venting pressure and material properties used by Verstraete et al. as was also found for the regional aircraft.

**Table 4:** Comparison of tank level performance for REG and LPA aircraft designed in this research and by Verstraete et al. [15] and SMR aircraft designed in this research and from Silberhorn et al. [19]. Note that fuel system mass was not reported by Verstraete et al

Parameters	REG		SMR		LPA	
	REG-LH2-a	Verstraete	SMR-LH2-b	Silberhorn	LPA-LH2-c	Verstraete
$m_\text {fuelSys}$ (kg)	–	–	749	744	–	–
$m_\text {fuel}$ (kg)	802	1150	5732	5985	23,294	40,000
$\eta _{\text {grav}}$	0.378	0.41	0.268	0.276	0.244	0.30

## Results

The objective of this research was to identify and compare possible solutions for the integration of $\text {L}\text {H}_\text {2}$ fuel system on turbine-powered airliners. Therefore, the design and analysis framework previously described was used to assess effects that different combinations of tank structure, fuselage diameter, tank layout, shape, venting pressure, and pressure control generate at aircraft level, depending on aircraft category. It was decided to compare kerosene and $\text {L}\text {H}_\text {2}$ aircraft for the same technology level and quantify the effect of the different energy carriers and associated integration. The chosen technology level is the one of current operational aircraft, as not to introduce assumptions on technological improvement factors, nor assumptions on how these improvements would alter the designs.

This section presents and discusses the $\text {L}\text {H}_\text {2}$ aircraft versions of REG turboprop, SMR turbofan, and LPA turbofan aircraft. For each aircraft category, one kerosene version and five $\text {L}\text {H}_\text {2}$ versions are reported for discussion, in an attempt to cover the investigated design space concisely. The first two $\text {L}\text {H}_\text {2}$ aircraft of each category serve to study the effect of the choice of tank structure (integral vs non-integral). The third $\text {L}\text {H}_\text {2}$ aircraft serves to explore the option of relocating the fuel such that it has no impact on the centre-of-gravity range. The fourth aircraft serves to analyse the effect of increasing the fuselage diameter (or equivalently the seats abreast). The fifth $\text {L}\text {H}_\text {2}$ aircraft allows for an investigation of different options for each aircraft category, e.g. a double-deck layout for LPA in order to reduce fuselage length.

The aircraft were designed for the top-level requirements of the ATR72-600, the A320neo and the A330-300 as specified in Table 1, only the fuel consumption differs, as explained in Sect. 2.5. Whereas both the kerosene and the $\text {L}\text {H}_\text {2}$ aircraft were designed for the same harmonic mission (maximum range with maximum payload), their payload-range diagrams are different, since the $\text {L}\text {H}_\text {2}$ tanks are sized (due to the thermodynamic limitations discussed in 2.3.3) for the harmonic mission only, as illustrated in Fig. 10. This design choice is further discussed later in the article, in Sect. 4.4.

The results’ tables (Tables 5, 6 and 7) report masses and drag contributions of those systems and components most affected by the change in fuel and tank integration choices, so that it can be understood what drives the differences in aircraft performance. In particular, three main aircraft performance parameters, namely MTOM, OEM and specific energy consumption (SEC), were selected as key performance indicators. MTOM and OEM are both relevant because they are directly used to size the engines, the wing (or the high-lift devices) and the landing gear, and because they are a good measure of an aircraft unit cost. The SEC directly impacts the operating costs and aircraft emissions. Naturally, through the cyclic effect in aircraft sizing, these three parameters influence each other. However, it is common to find studies where a hydrogen aircraft has a lower MTOM but higher OEM and SEC than its kerosene counterpart.

Extensive tables with data from less relevant components for comparison in the article are reported for completeness in Appendix A (see Table 9, Tables 10 and 11).

### Regional turboprop aircraft

**Fig. 11:**
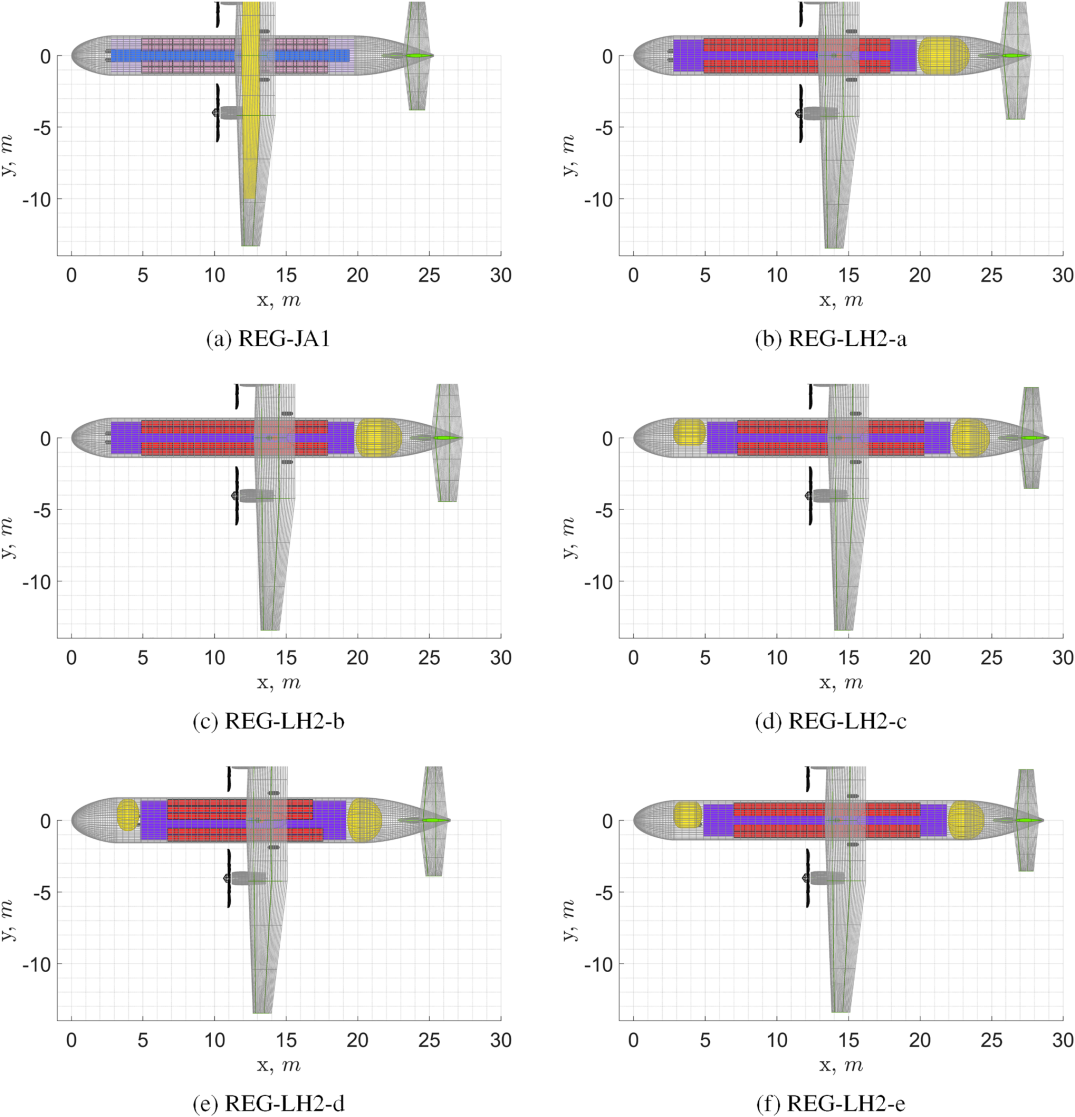
Top view of REG turboprop aircraft. See Table 5 for complementary data

The most relevant design parameters and design outputs of the baseline regional turboprop (REG) aircraft and of the $\text {L}\text {H}_\text {2}$ versions are presented in Table 5. Figure 11 shows top views of these aircraft. All presented versions use the venting pressure which was found to be optimal for the first aircraft version. Note that optimality of this venting pressure is not only dependent on performance of the tank insulator and structural shell materials, but also on tank design options and aircraft configuration, performance and mission.

A comparison between the **REG-LH2-a** and the **REG-LH2-b** shows that the use of an integral tank is slightly beneficial. Despite the tank mass being similar, its shorter length translates into a shorter fuselage, which brings mass and drag reductions. A comparative fuselage mass component breakdown shows that the **REG-LH2-b** has 5 *kg* lower skin mass, 15 *kg* lower stringers mass and 25 *kg* lower frame mass.

Comparison between the **REG-LH2-b** and the **REG-LH2-c** shows that a combination of an aft and a forward tank yields a similar aircraft mass and lower specific energy consumption. Note that the use of two tanks has a negative effect on the tank gravimetric index and on the combined-tanks length. However, it can be seen that despite the significant increase in the fuselage length, the fuselage mass of the **REG-LH2-c** is only slightly higher than the **REG-LH2-b**’s one. A comparative fuselage mass component breakdown shows the following: the **REG-LH2-c** has 30 *kg* higher crew floor mass (including cockpit-to-cabin corridor), 50 kg lower aft shell mass, 60 kg higher passengers’ cabin floor mass, 25 kg higher skin mass and 45 *kg* lower stringers mass. Overall, thanks also to a large reduction in horizontal tail mass, possible due to the smaller c.g. range, the OEM of the two aircraft remains similar. The specific energy consumption decreases, because the smaller centre-of-gravity shift, obtained by relocating the fuel centre of gravity, reduces both the trim drag and the parasite drag from the smaller horizontal tail now required.

Comparison between the **REG-LH2-d** and the **REG-LH2-c** shows that an increase in seats abreast from 4 to 5 slightly worsens the aircraft performance. The increase in fuselage mass is the main driver of this deterioration in performance. A comparative fuselage mass component breakdown shows the following: the **REG-LH2-d** has (due to the larger diameter) 10 kg higher crew floor mass, 30 kg lower windows mass, 10 kg higher wind shield mass, 25 kg higher cargo floor mass, 15 kg higher pressure bulkheads mass, 35 kg higher nose shell mass, 65 kg higher aft shell mass, 170 kg higher passengers’ cabin floor mass, 35 kg higher skin mass, 400 *kg* lower stringers mass and 115 kg higher frame mass. Note, however, that for 72 passengers the cabin space is not as efficiently used by the 5 seats abreast configuration as it is in the 4 seats abreast configuration. With a more refined design tool, the cabin space could be utilised better and a shorter aircraft could be obtained, reducing the difference in aircraft performance obtained here.

The fifth study, which is uniquely presented for this category, investigates the effect of using direct gas venting during the diversion and loiter phases. The direct venting option was chosen to be presented for this aircraft category because of its large reserve fuel fraction. Comparison between the **REG-LH2-e** and the **REG-LH2-c** shows that the use of direct venting reduces the insulation thickness and consequently the tank mass and length. This brings a slight improvement in all the main aircraft performance parameters. Note, however, that the total fuel mass, including the vented fuel mass, is larger for the **REG-LH2-e**, hence the SEC could be larger for this aircraft depending on the energy lost during hydrogen re-liquefaction. This also means that venting during the regular mission is, in terms of energy consumption, never advantageous.

With respect to the** REG-JA1** (kerosene baseline), the best performing $\text {L}\text {H}_\text {2}$ aircraft version, the **REG-LH2-e**, presents 10% higher OEM, similar MTOM and 3% higher SEC. The increase in OEM is caused by the heavier fuselage, by the addition of the $\text {L}\text {H}_\text {2}$ tanks, by the increase in the fuel system mass, by the increase in landing gear height and by the increase in wing mass. The lower MTOM is obtained thanks to the significantly lower fuel mass. The higher SEC is due to the higher average aircraft mass during flight. The higher parasite drag is here entirely due to the increase in fuselage size, whereas the parasite drag of the tail surfaces is reduced.^16^

**Table 5:** Input and output data for the REG turboprop aircraft. Parameters up to and including “Fuel fraction in aft tank” are input, the following are output, with the last three being the main performance parameters

Parameters	REG-JA1	REG-LH2-a	REG-LH2-b	REG-LH2-c	REG-LH2-d	REG-LH2-e
Tank structure	–	Non-integral	Integral	Int and non-int	Int and non-int	Int and non-int
Seats abreast EC	2-2	2-2	2-2	2-2	2-3	2-2
Cryotank layout	–	Aft	Aft	Aft and fwd	Aft and fwd	Aft and fwd
$P_{\text {vent}}$ (kPa)	–	300	300	300	300	300
Direct venting	–	no	no	no	no	yes
Fuel fraction in aft tank	–	1	1	0.75	0.75	0.8
$t_{\text {ins}}$ (mm) (aft)	0	99	97	106	104	74
$t_{\text {shell}_{\text {central}}}$ (mm) (aft)	0	2.0	2.1	2.1	2.4	2.1
$m_{\text {tank}}$ (kg) (aft+fwd)	0	303	288	315	333	287
$\eta _{\text {grav}}$ (aft+fwd)	0	0.378	0.361	0.401	0.415	0.368
$l_{\text {tank}}$ (m) (aft+fwd)	0	3.59	3.25	4.91	3.97	4.54
$m_{\text {fuel,vented}}$ (kg)	0	0	0	0	0	14
$m_{\text {fuelSys}}$ (kg)	210	494	494	492	494	491
$r_{\text {fus}}$ (m)	1.39	1.39	1.39	1.39	1.59	1.39
$l_{\text {fus}}$ (m)	25.3	27.7	27.3	29.0	26.5	28.6
*b* (m)	26.6	27.0	26.9	26.9	27.0	26.8
*S* (m$^2$)	59.1	60.5	60.3	60.3	60.5	59.9
MLM (t)	22.3	22.8	22.7	22.7	22.8	22.6
$\Delta x_{\text {c.g.}}$	0.330	0.600	0.585	0.273	0.311	0.280
$S_{ht}/S$	0.195	0.262	0.263	0.165	0.200	0.168
$m_{ht}$ (t)	0.14	0.21	0.21	0.12	0.15	0.12
$m_{\text {fus}}$ (t)	3.57	4.20	4.15	4.19	4.26	4.14
$m_{gear}$ (*t*)	0.78	0.82	0.81	0.87	0.78	0.86
$C_{D_{0,\text {ht}}}$ (count)	15	20	20	13	15	13
$C_{D_{0,\text {fus}}}$ (count)	66	71	70	74	77	74
$C_{D_{0,w}}$ (count)	76	76	76	76	75	76
$C_{D_{0}}$ (count)	208	216	216	213	217	212
$L/D_{\text {mid-cruise}}$	17.9	17.5	17.5	17.9	17.5	17.9
FM (t)	2.10	0.802 ($-$61.8%)	0.797 ($-$62%)	0.784 ($-$62.6%)	0.802 ($-$61.8%)	0.781 ($-$62.8%)
OEM (t)	13.2	14.7 (+11.5%)	14.6 (+10.8%)	14.6 (+10.8%)	14.7 (+11.4%)	14.5 (+9.9%)
MTOM (t)	22.8	23.0 (+1%)	22.9 (+0.5%)	22.9 (+0.5%)	23.0 (+1%)	22.8 ($-$0%)
SEC (kJ/pax/m)	1.01	1.07 (+5.8%)	1.07 (+5.2%)	1.05 (+3.3%)	1.07 (+5.8%)	1.04 (+2.9%)

### Short/medium range turbofan aircraft

**Fig. 12:**
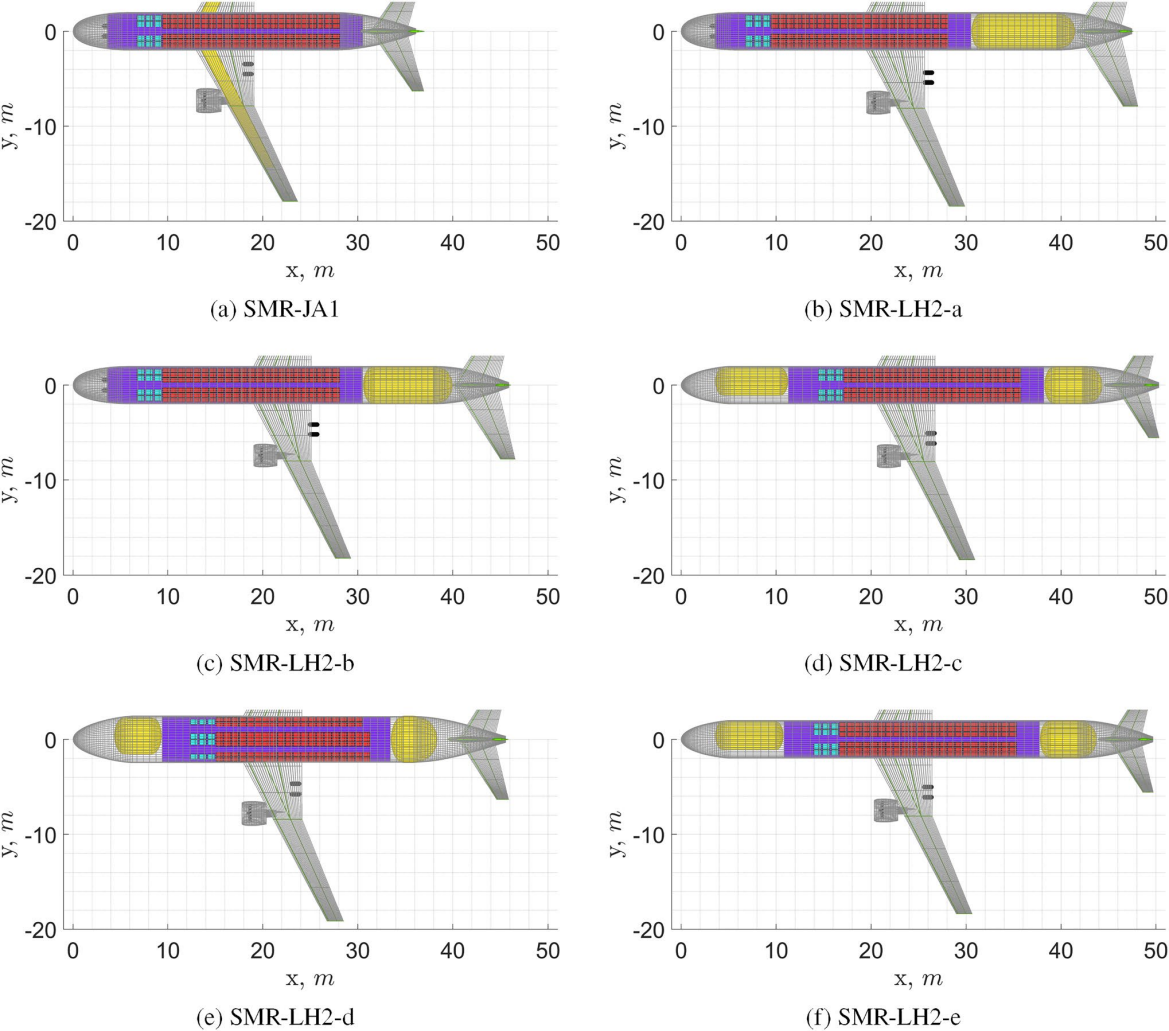
Top view of SMR turbofan aircraft. See Table 6 for complementary data

The most relevant design parameters and design outputs of the baseline short/medium-range turbofan (SMR) aircraft and of the $\text {L}\text {H}_\text {2}$ versions are presented in Table 6. Top views of these aircraft are shown in Fig. 12. All the presented versions employ the venting pressure which was found to be optimal for the first aircraft version.

Comparison between the **SMR-LH2-a** and the **SMR-LH2-b** concepts indicates that the use of an integral tank structure is beneficial in terms of both masses and specific energy consumption, similarly to the REG category but by a greater proportion. The latter effect is caused by the larger fuel mass fraction, which accentuates the benefits of using the integral tank structure.

The addition of a forward fuel tank that carries sufficient fuel to make the $\Delta x_{\text {c.g.}}$ fuel independent, has a positive effect on the specific energy consumption but a negative effect on the OEM and the MTOM, as can be concluded from the comparison between the **SMR-LH2-b** and **SMR-LH2-c** aircraft. Confronting this with what was seen for the correspondent versions of the REG aircraft, it can be noticed that the tank and fuselage mass penalties are more important than the horizontal tail mass saving, in absolute value, hence the worsening of OEM for this tank configuration, for the SMR category.

Comparison between the **SMR-LH2-d** and the **SMR-LH2-c** reveals that an increase in seats abreast from 6 to 7 has a large negative effect on the aircraft performance. Unlike for the REG aircraft, seats abreast can only be added following the addition of a second aisle. This means that only a relatively small reduction in fuselage length is obtained for a relatively large increase in fuselage diameter. The increase in fuselage mass and parasite drag are indeed larger (same coefficients, but larger reference area) than the one observed for the REG aircraft case.

The fifth version, the **SMR-LH2-e**, which is uniquely presented for this configuration, investigates the effect of using a higher venting pressure. The reduction in tank length with respect to the **SME-LH2-c**, obtained thanks to the lower insulation thickness required, entails a reduction in fuselage mass which more than compensates for the increase in tank mass due to the larger inner shell thickness. Notice how the shell thickness drives the tank mass while the insulation thickness is responsible for the tank length and consequently for the fuselage mass. This comparison shows that the venting pressure optimal for the aft tank layout is not necessarily also optimal for the aft-and-forward tank layout, not even with all other design variables being equal.

With respect to the **SMR-JA1** (kerosene baseline), the best performing $\text {L}\text {H}_\text {2}$ aircraft version, the **SMR-LH2-e**, presents 14% higher OEM, 4% lower MTOM and 3% higher SEC. The increase in OEM is caused by the heavier fuselage, by the addition of the $\text {L}\text {H}_\text {2}$ tanks, by the increase in the fuel system mass and by the increase in landing gear height. The MTOM is reduced due to the significantly lower fuel mass. The marginally higher SEC results from the slightly lower mid-cruise lift-to-drag ratio in combination with the slightly higher average aircraft mass during flight.

Note that the wing of the **SMR-LH2-c**, despite having a larger surface area than the kerosene baseline (because of the higher MLM), has the same mass. This is caused by the lower MTOM, which appears to determine the condition of critical loads during the wing structure sizing for both kerosene and $\text {L}\text {H}_\text {2}$ aircraft. The fuel bending relief available for kerosene aircraft proves to have a relatively small impact on the wing mass (in the order of 4%). This is understandable since the bending relief only has an impact on the ideal primary wing structure, which constitutes only about half of the wing mass. At this moment a gate constraint (36*m*) span was not enforced, as can be seen in the wing span reported in Table 6. However, for most of the solutions, the difference to the constraint is limited and the solution may be achievable by use of winglets instead of a span extension. These were not yet considered for the $\text {L}\text {H}_\text {2}$ designs. It does indicate that this is an important design criterion for this category of aircraft. The **SMR-LH2-d** is the most limiting and in fact already least likely design option given the specific cabin layout choice.

**Table 6:** Input and output data for the SMR turbofan aircraft. Parameters up to and including “Fuel fraction in aft tank” are input, the following are output, with the last three being the main performance parameters

Parameters	SMR-JA1	SMR-LH2-a	SMR-LH2-b	SMR-LH2-c	SMR-LH2-d	SMR-LH2-e
Tank structure	–	Non-integral	Integral	Int and non-int	Int and non-int	Int and non-int
Seats abreast EC	3-3	3-3	3-3	3-3	2-3-2	3-3
Cryotank layout	–	Aft	Aft	Aft and fwd	Aft and fwd	Aft and fwd
$P_\text {vent}$ (*kPa*)	–	250	250	250	250	300
Direct venting	–	No	No	No	No	No
Fuel fraction in aft tank	–	1	1	0.6	0.6	0.6
$t_{\text {ins}}$ (mm) (aft)	0	128	121	134	126	106
$t_{\text {shell}_{\text {central}}}$ (mm) (aft)	0	2.5	2.7	2.7	3.4	3.3
$m_{\text {tank}}$ (t) (aft+fwd)	0	1.73	1.54	1.77	1.81	1.80
$\eta _{\text {grav}}$ (aft+fwd)	0	0.294	0.268	0.313	0.294	0.321
$l_{\text {tank}}$ (m) (aft+fwd)	0	10.8	9.37	13.7	9.76	13.1
$m_{\text {fuelSys}}$ (kg)	280	753	749	746	762	746
$r_{\text {fus}}$ (m)	1.99	1.99	1.99	1.99	2.44	1.99
$l_{\text {fus}}$ (m)	36.1	47.5	45.9	50.3	45.5	49.7
*b* (m)	35.8	36.9	36.5	36.8	38.3	36.7
*S* (m$^2$)	122	130	127	129	140	129
MLM (t)	68.3	72.4	70.6	71.8	77.9	71.8
$\Delta x_{\text {c.g.}}$	0.172	0.605	0.565	0.118	0.124	0.117
$S_{ht}/S$	0.260	0.385	0.385	0.193	0.230	0.194
$m_{ht}$ (t)	1.18	2.02	1.96	0.86	1.18	0.87
$m_{fus}$ (t)	10.6	13.7	13.2	14.6	17.7	14.5
$m_{w}$ (t)	9.99	10.4	10.0	10.2	11.4	10.2
$m_{\text {gear}}$ (t)	2.67	3.20	3.05	3.43	3.35	3.4
$C_{D_{0,ht}}$ (count)	20	29	29	15	18	16
$C_{D_{0,\text {fus}}}$ (count)	60	73	73	78	78	77
$C_{D_{0}}$ (count)	212	234	233	225	225	224
$L/D_{\text {mid-cruise}}$	17.4	16.4	16.4	17.0	16.9	17.0
FM (t)	15.1	5.88 ($-$60.9%)	5.73 ($-$61.9%)	5.63 ($-$62.6%)	6.14 ($-$59.2%)	5.62 ($-$62.7%)
OEM (t)	44.8	51.4 (+14.8%)	49.8 (+11.1%)	51.0 (+13.9%)	56.9 (+27.1%)	50.9 (+13.7%)
MTOM (t)	79.1	76.6 ($-$3.2%)	74.8 ($-$5.5%)	75.9 ($-$4.1%)	82.4 (+4.1%)	75.9 ($-$4.2%)
SEC (kJ/pax/m)	0.778	0.842 (+8.2%)	0.821 (+5.5%)	0.806 (+3.7%)	0.878 (+13%)	0.804 (+3.3%)

### Long range turbofan aircraft

**Fig. 13:**
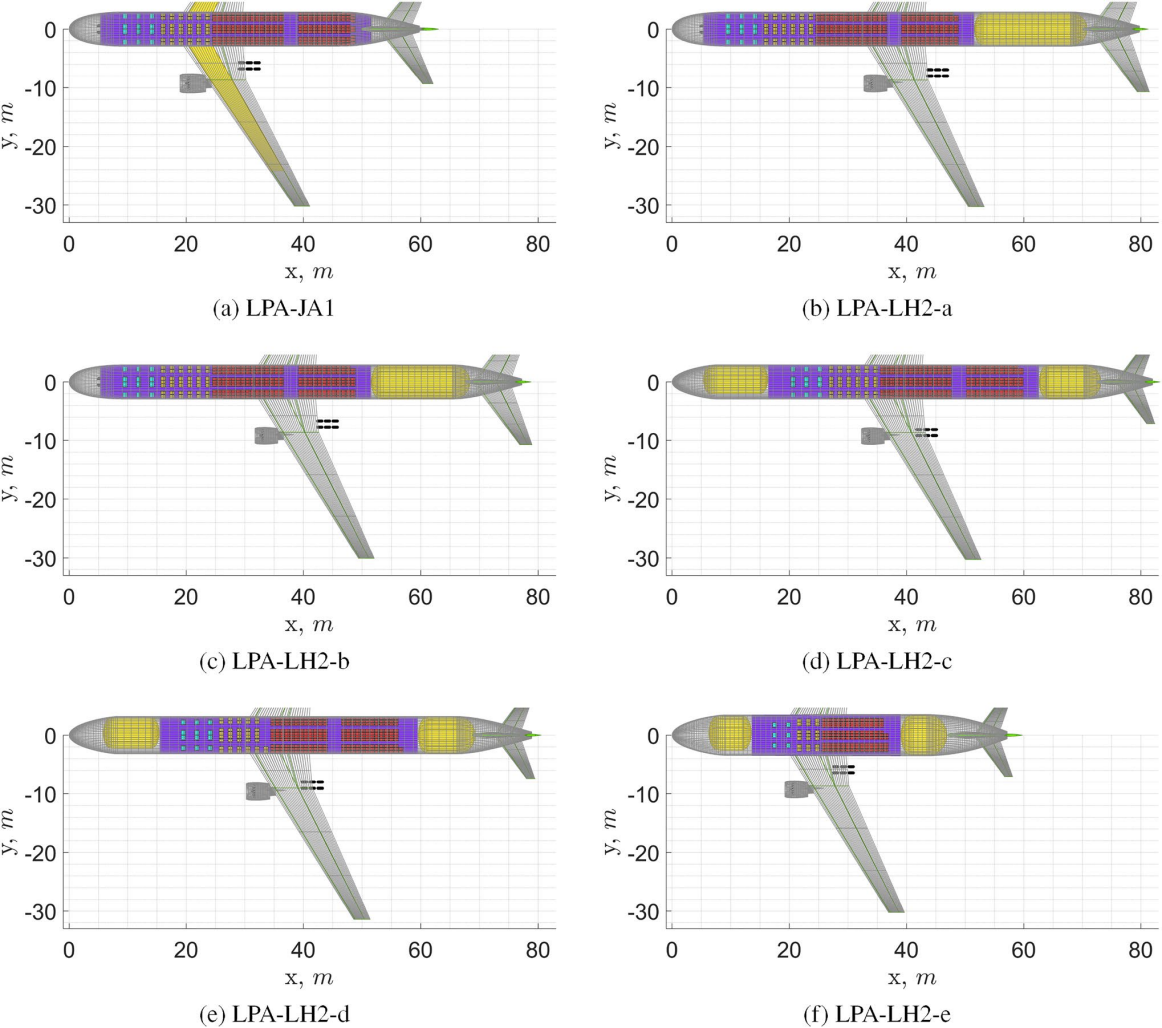
Top view of LPA turbofan aircraft. See Table 7 for complementary data

Table 7 presents the most relevant design parameters and outputs of the baseline long range turbofan (LPA) aircraft and of the $\text {L}\text {H}_\text {2}$ versions. Top views of these aircraft are shown in Fig. 13. The venting pressure used for the first four aircraft is the one that was found to be optimal for the first.

Examination of the **LPA-LH2-a** and **LPA-LH2-b** aircraft shows that the use of an integral tank structure is beneficial in terms of both masses and specific energy consumption, and by a similar amount to what was found for the SMR aircraft.

Comparison between the**LPA-LH2-b** and the **LPA-LH2-c** demonstrates that a combination of aft-and-forward tanks increases aircraft mass but significantly reduces specific energy consumption. The behaviour of the aircraft components’ masses is similar to what was observed for the SMR aircraft.

Increasing the seats abreast from 9 (**LPA-LH2-c**) to 10 (**LPA-LH2-d**) has a negative effect on the aircraft performance, almost as large as the one observed for the SMR aircraft. Note that as for the REG aircraft, seats abreast can be added without the addition of an aisle, hence the relative reduction in fuselage length and the relative increase in fuselage radius are approximately of the same magnitude. Unfortunately, the result of these changes is still an increase in both mass and parasite drag for the fuselage. Note, however, that a 3 *m* reduction in fuselage length, from 82 *m* to 79 *m* could be a very relevant advantage when considering the airport gate requirements, as these $\text {L}\text {H}_\text {2}$ LPA versions are longer than the current longest passenger aircraft (the 747-8, at 76.25 m) and some even exceed the 80 m box.

The fifth version, the **LPA-LH2-e**, which is uniquely presented for this configuration, investigates the effect of using a double-deck cabin, which should be able to more efficiently use the fuselage cross-section. The cabin layout of **LPA-LH2-c** is kept, with the passengers being equally split into two equally long and wide cabins (actual division has no impact on the sizing, hence an equal split is deemed justifiable for now). The tank gravimetric index remains similar, as the reduction in surface-to-volume ratio compensates for the larger circumferential stresses. The fuselage volume actually remains similar and the fuselage mass increases. Nevertheless, the fuselage wetted surface decreases, reducing the fuselage parasite drag. The landing gear mass decreases substantially, due to the much smaller strut height. The maximum container mass that can be carried decreases by 1 *t*, as less container fits in the cargo hold (they are still sufficient to store the required cargo—removing the same amount of containers would have no influence on fuselage length of single-deck aircraft). With respect to the **LPA-LH2-c**, the OEM and the MTOM remained similar while the SEC improves, thanks to the decrease in fuselage parasite drag.

With respect to the **LPA-JA1** (kerosene baseline), the best performing $\text {L}\text {H}_\text {2}$ aircraft version, the **LPA-LH2-c** (**LPA-LH2-e** is not considered here because of the significantly different configuration), presents 8% higher OEM, 15% lower MTOM and 4% lower SEC. The increase in OEM is caused by the larger fuselage, addition of $\text {L}\text {H}_\text {2}$ tanks, higher landing gear and the increase in fuel system mass. It is relieved by the reduction in the engine, wing, horizontal and vertical tail masses. The lower MTOM is obtained thanks to the significantly lower fuel mass. The lower SEC is due to the slightly lower mid-cruise lift-over-drag ratio in combination with the significantly lower average aircraft mass during flight. The higher parasite drag is entirely due to increased fuselage size, mitigated by the reduction in tail size. The same landing gear integration problem mentioned for the SMR aircraft affects the LPA aircraft too. **LPA-LH2-e** shows that a double-deck layout can be beneficial for these long range aircraft, considering the fuselage length. The integration of the main landing gear could pose some challenges, particularly for the version with an aft tank only (**LPA-LH2-a** and **LPA-LH2-b**), due to the aft positioning and the effect this would have on the yehudi. The main landing gear position has to be that aft to meet the tip-over constraint in high-fuel conditions. At this stage, this has not been considered and remains an area for further research.

**Table 7:** Input and output data for the LPA turbofan aircraft. Parameters up to and including “Fuel fraction in aft tank” are input, the following are output, with the last three being the main performance parameters

Parameters	LPA-JA1	LPA-LH2-a	LPA-LH2-b	LPA-LH2-c	LPA-LH2-d	LPA-LH2-e
Tank structure	–	Non-integral	Integral	Int and non-int	Int and non-int	Int and non-int
Seats abreast EC	3-3-3	3-3-3	3-3-3	3-3-3	3-4-3	3-3-3 twin$^{\mathrm{d}}$
Cryotank layout	–	Aft	Aft	Aft and fwd	Aft and fwd	Aft and fwd
$P_\text {vent}$ (kPa)	–	225	225	225	225	225
Direct venting	–	No	No	No	No	No
Fuel fraction in aft tank	–	1	1	0.6	0.6	0.6
$t_{\text {ins}}$ (mm) (aft)	0	136	131	142	136	136
$t_{\text {shell}_{\text {central}}}$ (mm) (aft)	0	3.4	3.6	3.6	4.0	4.4
$m_{\text {tank}}$ (t) (aft+fwd)	0	5.94	5.29	5.68	5.88	5.43
$\eta _{grav}$ (aft+fwd)	0	0.244	0.220	0.244	0.236	0.240
$l_{\text {tank}}$ (m) (aft+fwd)	0	19.2	16.8	21.6	19.4	15.1
$m_{\text {fuelSys}}$ (t)	0.407	1.08	1.08	1.07	1.09	1.06
$r_{\text {fus}}$ (m)	2.93	2.93	2.93	2.93	3.19	3.52
$l_{\text {fus}}$ (m)	59.8	79.8	77.2	82.0	78.9	57.2
*b* (m)	60.4	60.6	60.2	60.5	62.8	60.5
*S* (m$^2$)	362	364	358	363	391	362
MLM (*t*)	189	190	187	189	204	189
$\Delta x_{\text {c.g.}}$	0.287	0.657	0.650	0.190	0.158	0.076
$S_{ht}/S$	0.192	0.252	0.258	0.112	0.112	0.110
$m_{ht}$ (t)	2.48	3.37	3.43	1.25	1.37	1.25
$m_{\text {fus}}$ (t)	30.0	37.7	37.0	39.5	47.0	44.5
$m_{w}$ (t)	34.8	33.0	32.3	33.0	36.6	32.6
$m_{\text {gear}}$ (t)	11.1	12.4	12.0	13.2	13.8	9.6
$C_{D_{0,ht}}$ (count)	14	18	19	9	9	9
$C_{D_{0,\text {fus}}}$ (count)	46	61	60	62	60	53
$C_{D_{0}}$ (count)	178	196	195	187	184	174
$L/D_{\text {mid-cruise}}$	19.4	18.3	18.3	19	19.2	19.5
FM (t)	67.4	24.4 ($-$63.8%)	24.0 ($-$64.4%)	23.3 ($-$65.4%)	24.9 ($-$63.1%)	22.6 ($-$66.4%)
OEM (t)	129	139 (+7.8%)	136 (+5.7%)	138 (+7.6%)	153 (+18.8%)	138 (+7.6%)
MTOM (t)	242	209 ($-$13.6%)	205 ($-$14.9%)	207 ($-$14.2%)	223 ($-$7.6%)	207 ($-$14.5%)
SEC (kJ/pax/m)	0.113	0.113 (+0.2%)	0.111 ($-$1.3%)	0.108 ($-$4.2%)	0.115 (+2.3%)	0.105 ($-$7%)

^d^Double deck configuration

### Payload-range diagram and design considerations

This section discusses the choice of designing the fuel tanks of the $\text {L}\text {H}_\text {2}$ aircraft for the harmonic mission (maximum range with maximum payload) of the kerosene baseline and discusses the alternatives to this design choice. Kerosene aircraft suffer no significant performance penalties in offering the second segment in the payload-range diagram (between the harmonic and the maximum fuel mission), as they can trade payload mass for fuel mass, which can be stored in the wing. However, for an $\text {L}\text {H}_\text {2}$ aircraft, the amount of fuel stored directly determines the tank and the fuselage sizes due to the thermodynamic considerations in tank sizing (see 2.3.3). The addition of the same payload-range diagram segment, if the harmonic mission remains unchanged, would impose a significant penalty on aircraft performance.

It is worth showing what the effect of covering a larger portion of the payload-range diagram would be. As a case study the **SMR-JA1** aircraft is selected, being representative of the central size/range category analysed, and for the hydrogen version the **SMR-LH2-a** option was chosen, both because it is the simplest solution (see Sect. 4.2).

**Fig. 14:**
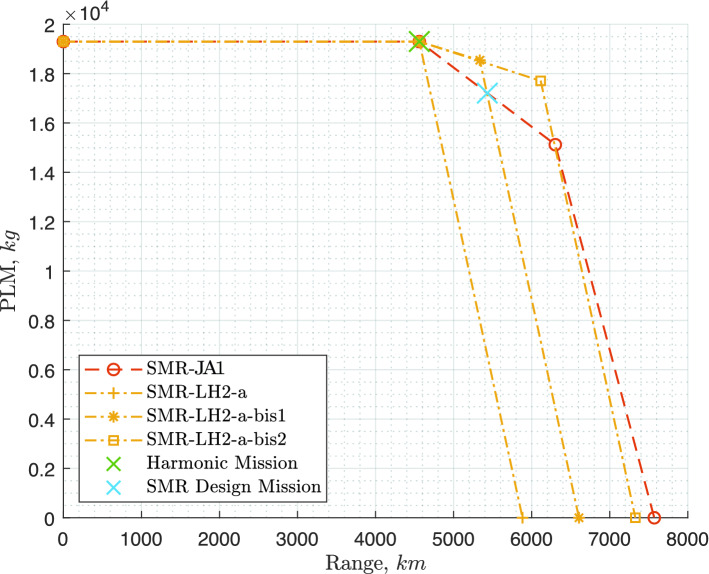
Payload-range diagrams of the SMR-JA1, the SMR-LH2-a, the SMR-LH2-a-bis1 and the SMR-LH2-a-bis2 aircraft

The payload-range diagrams of the **SMR-JA1** and the **SMR-LH2-a** aircraft are shown in Fig. 14, together with the payload-range diagrams of the **SMR-LH2-a-bis1** and **SMR-LH2-a-bis2**, which represent variants of the $\text {L}\text {H}_\text {2}$ designs that cover the payload-range diagram of the **SMR-JA1** design mission and the max fuel mission respectively. The design mission of the SMR-JA1 is chosen to be at a range halfway between the one of the harmonic mission and the one of the max fuel mission, a typical design mission for this type of aircraft.

Table 8 illustrates the effects of creating the second segment on the main performance parameters and on the aircraft components that are directly influenced by this design decision. Note that the penalty in performance increases exponentially with the maximum fuel mission range. Indeed, covering the **SMR-JA1** design mission, which has 19% longer range than the harmonic one, costs the **SMR-LH2-a-bis1** a 3% higher OEM, a 1% higher MTOM and a 2% higher SEC (evaluated at the harmonic mission). Covering the **SMR-JA1** maximum fuel mission, which has 38% higher range than the harmonic one, costs to the **SMR-LH2-a-bis2** a 11% higher OEM, a 7% higher MTOM and a 8% higher SEC (evaluated at the harmonic mission). This illustrates the sensitivity of $\text {L}\text {H}_\text {2}$ designs to the choice of design mission, much more than for traditional kerosene aircraft. Also note the span limit of 36 m, as for the other SMR aircraft would be violated if enforced. The same reasoning as for the SMR studies is followed here.

**Table 8:** Main aircraft performance parameters and aircraft components of the SMR-JA1, the SMR-LH2-a, the SMR-LH2-a-bis1 and the SMR-LH2-a-bis2 aircraft

Parameters	SMR-JA1	SMR-LH2-a	SMR-LH2-a-bis1	SMR-LH2-a-bis2
$m_{\text {tank}}$ (kg) (aft+fwd)	0	1730	2038	2456
$l_{\text {tank}}$ (m) (aft+fwd)	0	10.8	12.5	14.8
$l_{\text {fus}}$ (m)	36.1	47.5	49.2	51.4
*b* (m)	35.8	36.9	37.2	38.2
*S* (m$^2$)	122.3	129.7	132	138.8
$m_{\text {fus}}$ (t)	10.6	13.7	14.1	15.3
$m_{w}$ (t)	9.98	10.4	10.6	11.6
OEM (t)	44.8	51.5 (+15%)	52.8 (+17.9%)	56.4 (+26%)
MTOM (t)	79.1	76.7 ($-$3.1%)	77.6 (– 2%)	82.0 (+3.7%)
TOM (t) @ harm. miss	79.1	76.7 ($-$3.1%)	77.6 (– 2%)	82.0 (+3.7%)
SEC (kJ/pax/m) @ harm. miss	0.778	0.842 (+8.1%)	0.856 (+10%)	0.904 (+16.2%)
TOM (t) @ SMR des. miss	79.1	–	76.2 ($-$3.6%)	79.5 (+0.5%)
SEC (kJ/pax/m) @ SMR des. miss	0.762	–	0.834 (+9.4%)	0.861 (+13%)

## Conclusion

This research investigated and compared possible solutions to the integration of liquid hydrogen fuel system on REG, SMR and LPA turbine-powered airliners. The results are not limited only to the variation in the top-level aircraft performance parameters but also the relative changes in mass and drag contributions of those individual aircraft components affected by the change in fuel. This approach was used to gain a deeper understanding of the reasons why the optimal tank integration strategy seems to depend, in literature, on aircraft range category. However, the current study did not find such significant differences, indicating that differences are actually driven by the various design choices (e.g. mission, cabin layout or material properties) used in literature studies. It was decided to compare kerosene and $\text {L}\text {H}_\text {2}$ aircraft for the same technology level and quantify the effect of the different energy carriers and associated integration. The chosen technology level is the one of current operational aircraft, as not to introduce assumptions on technological improvements factors, and assumptions on how these improvements would alter the designs.

**Fig. 15:**
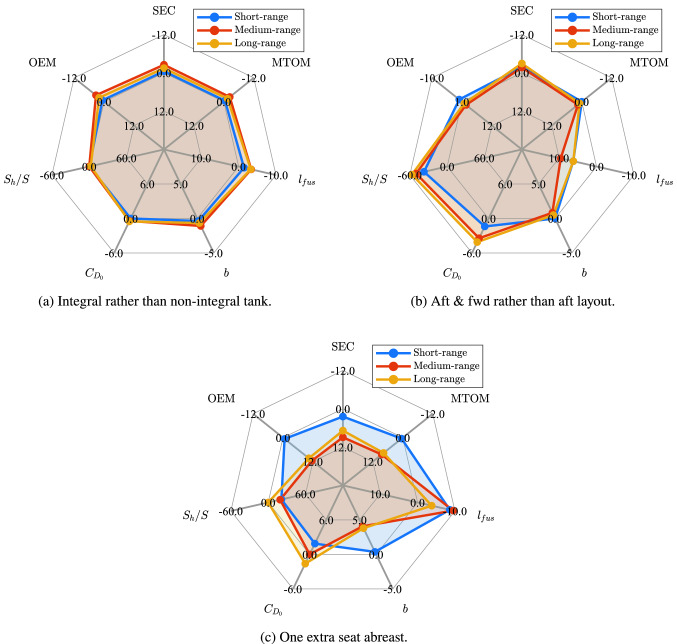
Effect of aircraft range category on performance parameters. The values represent the parameters changes, in percentage, between the $\text {L}\text {H}_\text {2}$ versions which differ only in the indicated design choice

Nevertheless, the created design and analysis framework proved to be capable of appreciating the effects that different combinations of tank structure, fuselage diameter, tank layout, shape, venting pressure, and pressure control generate at both the systems and aircraft levels, for the different aircraft categories. To summarise and visualise how the effects of the design choices for hydrogen tank integration vary with aircraft category, the relative performance changes are presented in Fig. 15.

With respect to the choice of using an integral rather than a non-integral tank structure, it can be seen in Fig. 15a that the benefits increase in magnitude with aircraft category (aircraft range and payload being the key differentiating factors between categories). Concerning the choice of using an aft-and-forward rather than an aft tank layout, Fig. 15b shows that the short/medium-range and the large passenger aircraft are affected the most. They both gain in specific energy consumption and lose in operational empty mass and maximum take-off mass. When considering the choice of increasing the fuselage diameter by adding one seat abreast, it can be seen that the short/medium-range aircraft suffers the most due to the required addition of an aisle. The large passenger aircraft suffers smaller penalties and the regional one is rather unaffected by the change (Fig. 15b). It was also concluded that direct venting has, when done efficiently, a small positive effect, that the optimal venting pressure varies with the aircraft configuration, performance, and mission. Furthermore, the use of a double-deck cabin can be used to reduce the large fuselage length, without suffering the large performance degradation related to an increase in fuselage radius. Finally, an investigation of the effect of sizing the tank for missions longer than the harmonic mission, to better cover the payload-range capabilities of the kerosene baseline aircraft, showed the exponential increase in masses and specific energy consumption penalties with increasing mission range.

The first recommendation for future work concerns the landing gear sizing and integration. The large shift in centre of gravity, encountered when using a single aft layout tank, creates the following two problems for the landing gear: first, the main landing gear presents a structural attachment challenge, as in order to prevent tip-over at take-off (especially in low payload configuration) and allow an acceptable scrape angle, the landing gear has to be positioned very aft with respect to the wing structure. Hence, this challenge impacts the design. Second, the large centre-of-gravity range means that the load on the nose gear will vary considerably depending on the aircraft loading (fuel, passengers, and cargo). Therefore, we recommended to pay extra attention to landing gear design for $\text {L}\text {H}_\text {2}$ aircraft.

A second recommendation addresses the cabin layout. The increase in seats abreast at constant passenger number makes the use of the cabin floor less efficient when using the method employed in this research. In reality, that cabin space could be optimised by rearranging the galleys and lavatories to use the space left by the incomplete seat rows. Alternatively, when not comparing aircraft with identical passenger capacity, the total number of seats could be adjusted to complete every row.

## Extensive comparison tables

This appendix contain extensive tables (Tables 9, 10 and 11) with the results of the investigated aircraft.

**Table 9:** Input and output data for the REG turboprop aircraft. Parameters up to and including “Fuel fraction in aft tank” are input, the following are output, with the last three being the KPIs

Parameters	REG-JA1	REG-LH2-a	REG-LH2-b	REG-LH2-c	REG-LH2-d	REG-LH2-e
Tank structure	–	Non-integral	Integral	Int and non-int	Int and non-int	Int and non-int
Seats abreast EC	2-2	2-2	2-2	2-2	2-3	2-2
Cryotank layout	–	Aft	Aft	Aft and fwd	Aft and fwd	Aft and fwd
$P_{\text {vent}}$ (kPa)	–	300	300	300	300	300
Direct venting	–	No	No	No	No	Yes
Fuel fraction in aft tank	–	1	1	0.75	0.75	0.80
$t_{\text {ins}}$ (mm) (aft)	0	99	97	106	104	74
$t_{\text {shell}_{\text {central}}}$ (mm) (aft)	0	2.0	2.1	2.1	2.4	2.1
$m_{\text {tank}}$ (kg) (aft+fwd)	0	303	288	315	333	287
$\eta _{\text {grav}}$ (aft+fwd)	0	0.378	0.361	0.401	0.415	0.368
$r_{\text {tank}}$ (m) (aft)	0	1.39	1.39	1.39	1.59	1.39
$l_{\text {tank}}$ (m) (aft+fwd)	0	3.59	3.25	4.91	3.97	4.54
$m_{\text {fuel,unusable}}$ (kg)	0	44	43	43	44	43
$m_{\text {fuel,vented}}$ (kg)	0	0	0	0	0	14
$m_{\text {fuelSys}}$ (kg)	210	494	494	492	494	491
$r_{\text {fus}}$ (m)	1.39	1.39	1.39	1.39	1.59	1.39
$l_{\text {fus}}$ (m)	25.3	27.7	27.3	29.0	26.5	28.6
*b* (m)	26.6	27.0	26.9	26.9	27.0	26.8
*S* (m$^2$)	59.1	60.5	60.3	60.3	60.5	59.9
MLM (t)	22.3	22.8	22.7	22.7	22.8	22.6
*W*/*S* (kN/m$^2$)	0.378	0.373	0.373	0.373	0.373	0.373
*W*/*P* (N/W)	0.0613	0.0609	0.0610	0.0611	0.0606	0.0611
$\Delta x_{\text {c.g.}}$	0.330	0.600	0.585	0.273	0.311	0.280
$S_{ht}/S$	0.195	0.262	0.263	0.165	0.200	0.168
$m_{vt}$ (*t*)	0.14	0.14	0.14	0.12	0.14	0.12
$m_{ht}$ (t)	0.14	0.21	0.21	0.12	0.15	0.12
$m_{\text {fus}}$ (t)	3.57	4.20	4.15	4.19	4.26	4.14
$m_{w}$ (t)	2.24	2.35	2.34	2.32	2.35	2.31
$m_{\text {engines}}$ (t)	1.62	1.63	1.63	1.63	1.64	1.62
$m_{\text {gear}}$ (t)	0.78	0.82	0.81	0.87	0.78	0.86
$m_{\text {furnishing}}$ (t)	1.37	1.37	1.37	1.37	1.39	1.37
$C_{D_{0,vt}}$ (count)	14	13	14	13	14	13
$C_{D_{0,ht}}$ (count)	15	20	20	13	15	13
$C_{D_{0,\text {fus}}}$ (count)	66	71	70	74	77	74
$C_{D_{0,w}}$ (count)	76	76	76	76	75	76
$C_{D_{0}}$ (count)	208	216	216	213	217	212
$L/D_{\text {mid-cruise}}$	17.9	17.5	17.5	17.9	17.5	17.9
FM (t)	2.10	0.802 ($-$61.8%)	0.797 (-62%)	0.784 ($-$62.6%)	0.802 ($-$61.8%)	0.781 ($-$62.8%)
OEM (t)	13.2	14.7 (+11.5%)	14.6 (+10.8%)	14.6 (+10.8%)	14.7 (+11.4%)	14.5 (+9.9%)
MTOM (t)	22.8	23.0 (+1%)	22.9 (+0.5%)	22.9 (+0.5%)	23.0 (+1%)	22.8 (-0%)
SEC (kJ/pax/m)	1.01	1.07 (+5.8%)	1.07 (+5.2%)	1.05 (+3.3%)	1.07 (+5.8%)	1.04 (+2.9%)

**Table 10:** Input and output data for the SMR turbofan aircraft. Parameters up to and including “Fuel fraction in aft tank” are input, the following are output, with the last three being the KPIs

Parameters	SMR-JA1	SMR-LH2-a	SMR-LH2-b	SMR-LH2-c	SMR-LH2-d	SMR-LH2-e
Tank structure	–	Non-integral	Integral	Int and non-int	Int and non-int	Int and non-int
Seats abreast EC	3-3	3-3	3-3	3-3	2-3-2	3-3
Cryotank layout	–	Aft	Aft	Aft and fwd	Aft and fwd	Aft and fwd
$P_\text {vent}$ (kPa)	–	250	250	250	250	300
Direct venting	–	No	No	No	No	No
Fuel fraction in aft tank	–	1	1	0.6	0.6	0.6
$t_{\text {ins}}$ (mm) (aft)	0	128	121	134	126	106
$t_{\text {shell}_{\text {central}}}$ (mm) (aft)	0	2.5	2.7	2.7	3.4	3.3
$m_{\text {tank}}$ (t) (aft+fwd)	0	1.73	1.54	1.77	1.81	1.80
$\eta _{\text {grav}}$ (aft+fwd)	0	0.294	0.268	0.313	0.294	0.321
$r_{\text {tank}}$ (m) (aft)	0	1.86	1.99	1.99	2.44	1.99
$l_{\text {tank}}$ (m) (aft+fwd)	0	10.8	9.37	13.7	9.76	13.1
$m_{\text {fuel,unusable}}$ (kg)	0	269	262	257	281	305
$m_{\text {fuelSys}}$ (kg)	280	753	749	746	762	746
$r_{\text {fus}}$ (m)	1.99	1.99	1.99	1.99	2.44	1.99
$l_{\text {fus}}$ (m)	36.1	47.5	45.9	50.3	45.5	49.7
*b* (m)	35.8	36.9	36.5	36.8	38.3	36.7
*S* (m$^2$)	122	130	127	129	140	129
MLM (t)	68.3	72.4	70.6	71.8	77.9	71.8
*W*/*S* (kN/m$^2$)	6.35	5.79	5.79	5.78	5.79	5.78
*T*/*W*	0.310	0.293	0.293	0.292	0.293	0.292
$\Delta x_{\text {c.g.}}$	0.172	0.605	0.565	0.118	0.124	0.117
$S_{ht}/S$	0.260	0.385	0.385	0.193	0.230	0.194
$m_{vt}$ (t)	0.54	0.46	0.46	0.42	0.53	0.43
$m_{ht}$ (t)	1.18	2.02	1.96	0.86	1.18	0.87
$m_{\text {fus}}$ (t)	10.6	13.7	13.2	14.6	17.7	14.5
$m_{w}$ (t)	9.99	10.4	10.0	10.2	11.4	10.2
$m_{\text {engines}}$ (t)	7.66	7.00	6.84	6.93	7.52	6.93
$m_{\text {gear}}$ (t)	2.67	3.20	3.05	3.43	3.35	3.40
$m_{\text {furnishing}}$ (t)	3.60	3.68	3.64	3.64	4.04	3.64
$C_{D_{0,vt}}$ (count)	12	10	10	9	10	9
$C_{D_{0,ht}}$ (count)	20	29	29	15	18	16
$C_{D_{0,\text {fus}}}$ (count)	60	73	73	78	78	77
$C_{D_{0,w}}$ (count)	67	68	68	68	65	68
$C_{D_{0}}$ (count)	212	234	233	225	225	224
$L/D_{\text {mid-cruise}}$	17.4	16.4	16.4	17	16.9	17
FM (t)	15.1	5.88 ($-$60.9%)	5.73 ($-$61.9%)	5.63 ($-$62.6%)	6.14 ($-$59.2%)	5.62 ($-$62.7%)
OEM (t)	44.8	51.4 (+14.8%)	49.8 (+11.1%)	51.0 (+13.9%)	56.9 (+27.1%)	50.9 (+13.7%)
MTOM (t)	79.1	76.6 ($-$3.2%)	74.8 ($-$5.5%)	75.9 ($-$4.1%)	82.3 (+4.1%)	75.9 ($-$4.2%)
SEC (kJ/pax/m)	0.778	0.842 (+8.2%)	0.821 (+5.5%)	0.806 (+3.7%)	0.878 (+13%)	0.804 (+3.3%)

**Table 11:** Input and output data for the LPA turbofan aircraft. Parameters up to and including “Fuel fraction in aft tank” are input, the following are output, with the last three being the KPIs

Parameters	LPA-JA1	LPA-LH2-a	LPA-LH2-b	LPA-LH2-c	LPA-LH2-d	LPA-LH2-e
Tank structure	–	Non-integral	Integral	Int and non-int	Int and non-int	Int and non-int
Seats abreast EC	3-3-3	3-3-3	3-3-3	3-3-3	3-4-3	3-3-3 twin$^{\mathrm{e}}$
Cryotank layout	–	Aft	Aft	Aft and fwd	Aft and fwd	Aft and fwd
$P_\text {vent}$ (*kPa*)	–	225	225	225	225	225
Direct venting	–	No	No	No	No	No
Fuel fraction in aft tank	–	1	1	0.6	0.6	0.6
$t_{\text {ins}}$ (mm) (aft)	0	136	131	142	136	136
$t_{\text {shell}_{\text {central}}}$ (mm) (aft)	0	3.4	3.6	3.6	4.0	4.4
$m_{\text {tank}}$ (t) (aft+fwd)	0	5.94	5.29	5.68	5.88	5.43
$\eta _{\text {grav}}$ (aft+fwd)	0	0.244	0.22	0.244	0.236	0.24
$r_{\text {tank}}$ (m) (aft)	0	2.75	2.93	2.93	3.19	3.52
$l_{\text {tank}}$ (m) (aft+fwd)	0	19.2	16.8	21.6	19.4	15.1
$m_{\text {fuel,unusable}}$ (t)	0	1.01	0.994	0.965	1.03	0.937
$m_{\text {fuelSys}}$ (t)	0.407	1.08	1.08	1.07	1.09	1.06
$r_{\text {fus}}$ (m)	2.93	2.93	2.93	2.93	3.19	3.52
$l_{\text {fus}}$ (m)	59.8	79.8	77.2	82.0	78.9	57.2
*b* (m)	60.4	60.6	60.2	60.5	62.8	60.5
*S* (m$^2$)	362	364	358	363	391	362
MLM (t)	189	190	187	189	204	189
*W*/*S* (kN/m$^2$)	0.655	0.563	0.563	0.561	0.560	0.559
*T*/*W*	0.266	0.246	0.246	0.242	0.243	0.245
$\Delta x_{\text {c.g.}}$	0.287	0.657	0.650	0.190	0.158	0.076
$S_{ht}/S$	0.192	0.252	0.258	0.112	0.112	0.110
$m_{vt}$ (t)	1.68	1.33	1.36	1.23	1.46	1.80
$m_{ht}$ (t)	2.48	3.37	3.43	1.25	1.37	1.25
$m_{fus}$ (t)	30.0	37.7	37.0	39.5	47.0	44.5
$m_{w}$ (t)	34.8	33.0	32.3	33.0	36.6	32.6
$m_{\text {engines}}$ (t)	18.8	14.9	14.7	14.6	15.8	14.7
$m_{\text {gear}}$ (t)	11.1	12.4	12.0	13.2	13.8	9.62
$m_{\text {furnishing}}$ (t)	9.58	9.67	9.66	9.66	10.3	9.55
$C_{D_{0,vt}}$ (count)	11	9	10	9	9	12
$C_{D_{0,ht}}$ (count)	14	18	19	9	9	9
$C_{D_{0,fus}}$ (count)	46	61	60	62	60	53
$C_{D_{0,w}}$ (count)	62	63	63	63	62	60
$C_{D_{0}}$ (count)	178	196	195	187	184	174
$L/D_{\text {mid-cruise}}$	19.4	18.3	18.3	19	19.2	19.5
FM (t)	67.4	24.4 ($-$63.8%)	24.0 ($-$64.4%)	23.3 ($-$65.4%)	24.9 ($-$63.1%)	22.6 ($-$66.4%)
OEM (t)	129	139 (+7.8%)	136 (+5.7%)	138 (+7.6%)	153 (+18.8%)	138 (+7.6%)
MTOM (t)	241	209 ($-$13.6%)	205 ($-$14.9%)	207 ($-$14.2%)	223 ($-$7.6%)	207 ($-$14.5%)
SEC (kJ/pax/m)	0.113	0.113 (+0.2%)	0.111 ($-$1.3%)	0.108 ($-$4.2%)	0.115 (+2.3%)	0.105 (– 7%)

^e^Double deck configuration

## Latin symbols

*A*: Outer tank surface (m$^2$)
*b*: Span (m)
$C_{D_{0}}$: Aircraft parasite drag coefficient (−)
$e_w$: Safety factor for joint efficiency in ASME Code (−)
*g*: Gravitational acceleration (m/s$$^2$$)
$h_{lg}$: Latent heat of vaporisation (J/kg)
*K*: Coefficient for the linear acceleration (−)
$k_{fs}$: Fuel system fraction (−)
$k_\text {ins}$: Effective thermal conductivity of the insulation material (W/(mK))
*L*/*D*: Lift over drag ratio (−)
*l*: Length (m)
*m*: Mass (kg)
$\dot{m}$: Mass flow rate (kg/s)
$N_e$: Number of engines (−)
$N_{ft}$: Number of fuel tanks (−)
*P*: Pressure (Pa)
$\dot{Q}_w$: Tank heating rate (W)
*r*: Radius (*m*)
*S*: Wing surface area (m$^2$)
*T*: Temperature (K)
*t*: Time (s)
$$t_{..}$$: Thickness (m)
$$t_{r}$$: Taper ratio (−)
*T*/*W*: Trust loading (−)
*u*: Specific internal energy of tank fluid (J/kg)
*V*: Tank fluid (liquid and vapour) volume (m$^3$)
$$V_{ft}$$: Fuel volume (*L*)
$\dot{W}_\text {mix}$: Rate of work done on the fluid (W)
*W*/*P*: Power loading (N/W)
*W*/*S*: Wing loading (N/m$$^2$$)

## Greek symbols

$\Delta x_{c.g.}$: Center of gravity range normalised with MAC (−)
$\Delta P_{hydrost}$: Hydrostatic pressure increment from aircraft accelerations (Pa)
$\eta _{\text {grav}}$: Tank gravimetric index ($m_\text {tank}/m_\text {fuel}$)
$\Lambda _{0.5}$: Half chord sweep angle (degrees)
$$\rho$$: Density (kg/m$$^3$$)
$\sigma$: Allowable stress of tank inner shell material (Pa)
$\phi$: Hydrogen energy derivative (Pa $\cdot \text {m}^3/\text {J}$)

## Subscripts

al: Aluminium - 2219-T851 alloy
amb: Ambient
cabin: Cabin
central: Central tank section
fuel: Fuel
fuelSys: Fuel system
fus: Fuselage
g: Gaseous
ht: Horizontal tail
ins: Insulation—polyurethane
l: Liquid
max: Maximum
mean: Mean
mid-cruise: Mid-cruise
min: Minimum
out: Outside of tank
shell: Structural (or inner) shell
tank: Tank
vent: Venting
vt: Vertical Tail
w: Wing

## Abbreviations

ATAG: Air Transport Action Group
BSFC: Brake-specific fuel consumption (g/(kWh))
ESDU: Engineering sciences data unit
$$\text {G}\text {H}_\text {2}$$: Gaseous hydrogen
ICAO: International Civil Aviation Organization
ISA: International Standard Atmosphere
$$\text {L}\text {H}_\text {2}$$: Liquid hydrogen
LPA: Large passenger aircraft
MAC: Mean aerodynamic chord (m)
MLM: Maximum landing mass (kg)
MTOM: Maximum take-off mass (kg)
MZFM: Maximum zero fuel mass (kg)
OEM: Operational empty mass (kg)
PLM: Payload mass (kg)
REG: Regional
SEC: Specific energy consumption (on standard mission) (J/pax/m)
SMR: Short/medium range
TSFC: Thrust-specific fuel consumption (kg/(Ns))

## Footnotes

1 Waypoint 2050. Balancing growth in connectivity with a comprehensive global air transport response to the climate emergency. Tech. rep., Air Transport Action Group, Sep. 2020.

2 Hydrogen-powered aviation A fact-based study of hydrogen technology, economics, and climate impact by 2050, Tech. rep., Clean Sky 2 JU and FCH 2 JU, May 2020.

3 Air transport, passengers carried, https://data.worldbank.org/indicator/IS.AIR.PSGR, Retrieved on 03/12/2020.

4 Aircraft Technology Roadmap to 2050, Tech. rep., IATA, 2018.

5 Peter, H., Forever fuel: the story of hydrogen, Westview Press, 1981.

6 Liquid Hydrogen as a Propulsion Fuel, 1945–1959. Bee Project, https://history.nasa.gov/SP-4404/ch6-4.htm. Retrieved on 03/12/2020.

7 Liquid Hydrogen as a Propulsion Fuel, 1945-1959. Suntan, https://www.hq.nasa.gov/office/pao/History/SP-4404/ch8-1.htm. Retrieved on 03/12/2020.

8 Faaß R., Cryoplane, https://www.fzt.haw-hamburg.de/pers/Scholz/dglr/hh/text_2001_12_06_Cryoplane.pdf, 2001. Retrieved on 04/12/2020.

9 Liquid hydrogen-fuelled aircraft – system analysis, Tech. Rep. GRD1-1999-10014, Airbus Deutschland GmbH,Sep 2003.

10 Airbus, ZEROe, https://www.airbus.com/en/innovation/zero-emission/hydrogen/zeroe. Retrieved on 01/04/2022.

11 Airbus, Introducing ZEROe, https://www.youtube.com/watch?v=525YtyRi_Vc, 2020. Retrieved on 04/12/2020.

12 Tupolev Tu-155, https://avia-pro.net/blog/tu-155 (2015), retrieved on 17/12/2020.

13 When using the word counterpart, it is meant that the aircraft is designed for the same mission (same payload /number of passengers and same range).

14 This image illustrates the fuselage parameterisation, the aircraft shown here have identical radii and, therefore, the same nose-cone and cockpit lengths. The nose shape is different because of the way the curve that is used to visualise the aircraft shape is plotted.

15 The maximum US Military Standard 210 (MIL 210) hot day temperature is 312.6 K ($39.5^{\circ }\hbox {C}$) at sea level.

16 The transparency of the cabin floor and seats shows the hidden main landing gear wheels. 4 main gear wheels are also used for the hydrogen aircraft versions.
